# Study of the Photophysical Properties and the DNA Binding of Enantiopure [Cr(TMP)_2_(dppn)]^3+^ Complex

**DOI:** 10.1021/acs.inorgchem.4c03590

**Published:** 2024-12-03

**Authors:** Daniel Graczyk, Rory A. Cowin, Dimitri Chekulaev, Maisie A. Haigh, Paul A. Scattergood, Susan J. Quinn

**Affiliations:** † School of Chemistry, University College Dublin, Dublin 4 D04 V1W8, Ireland; ‡ Department of Chemistry, University of Sheffield, Brook Hill, Sheffield S1 3HF, U.K.; § Department of Chemistry, School of Applied Sciences, University of Huddersfield, Queensgate, Huddersfield HD1 3DH, U.K.

## Abstract

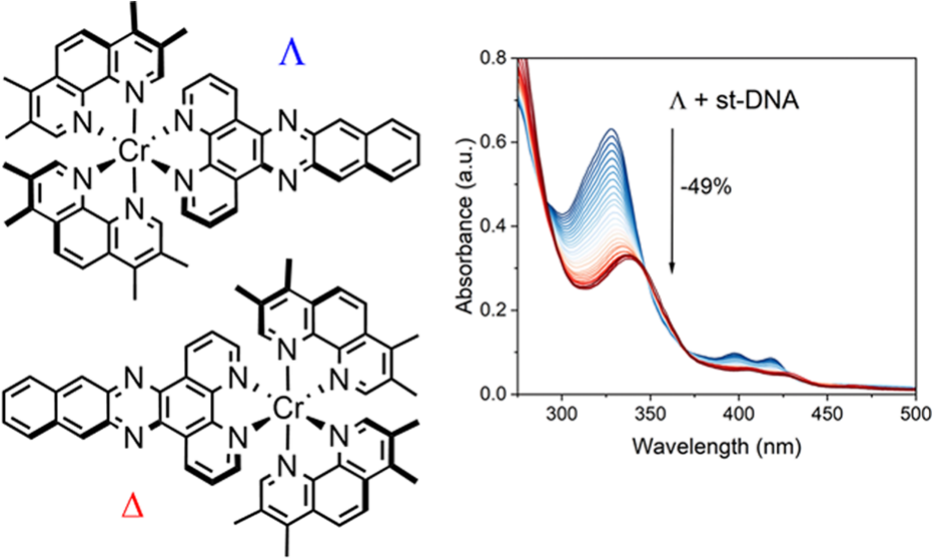

The preparation, electrochemistry and photophysical properties of a heteroleptic chromium(III) polypyridyl complex *rac-*[Cr(TMP)_2_(dppn)]^3+^ (**1**) containing two 3,4,7,8-tetramethyl-1,10-phenanthroline (TMP) ligands and the π-extended benzodipyrido[3,2-a:2′,3′-*c*]phenazine (dppn) ligand are reported. The visible absorption spectrum of **1** reveals distinct bands between 320 and 420 nm characteristic of dppn-based ligand-centered transitions, with **1** found to be nonemissive in aqueous solution but weakly luminescent in aerated acetonitrile solution. Transient visible absorption (TrA) spectroscopy reveals that 400 nm excitation of **1** leads to initial population of a ligand-to-metal charge transfer (LMCT) state which evolves within tens of ps to form a dppn-localized intraligand (^3^IL) state which persists for longer than 7 ns and efficiently sensitizes singlet oxygen. Chiral resolution and DNA binding of the lambda and delta enantiomers of **1** to four different DNA systems is reported. In all cases the lambda enantiomer shows greater affinity for DNA and in particular AT-rich DNA. Thermal denaturation reveals that the lambda enantiomer stabilizes the DNA more. There is also a greater stabilization of the AT-containing DNA sequences compared to GC DNA.

## Abstract 2

The synthesis and characterization of a novel Cr(III) polypyridyl complex, [Cr(TMP)_2_(dppn)]^3+^, are reported. Spectroscopic measurements reveal the dominance of dppn-based states on the photophysical properties of the complex, with a long-lived triplet intraligand state capable of sensitizing singlet oxygen. Chiral resolution of the complex affords the lambda and delta enantiomers; the former is shown to bind and stabilize DNA to a greater extent, with preference for AT-rich DNA systems.

## Introduction

Transition metal polypyridyl complexes are increasingly being studied as alternatives to porphyrins for light-activated therapeutic applications.^1−6^ Their versatile photophysical properties give rise to a range of phototherapeutic modes of action that include direct electron transfer leading to photo-oxidation of guanine,^7−9^ ligand ejection and adduct formation,^10,11^ and the most readily studied, Type I and Type II photodynamic therapy (PDT) processes.^12^ In the latter instance, Type I PDT involves electron transfer to form reactive oxygen radical species, while Type II involves energy transfer to molecular oxygen to form the cytotoxic singlet oxygen species (^1^O_2_). Extended polypyridyl ligands have attracted attention as they support intraligand charge transfer (ILCT) transitions that lead to the formation of long-lived triplet excited states, which can participate in Type I or Type II processes, though Type II processes tend to be more common. An excellent example is the Ru(II) polypyridyl complex TLD1433, which has entered phase II clinical trial for treating high-risk nonmuscle invasive bladder cancer.^2,13^ In TLD1433 the extended conjugation on the α-terthienyl-appended ligand supports a long-lived triplet intraligand (^3^IL) charge transfer excited state, which acts as a highly effective sensitizer of ^1^O_2_ through a Type II process, and can also participate in Type I electron transfer reactions.^3,4^ Another π-extended ligand that has received attention is benzo[*i*]dipyrido[3,2-a:2′,3′-*c*]phenazine (dppn), whose complexes readily form long-lived triplet states upon visible light excitation.^14,15^

Extended polypyridyl ligands also serve to enhance intercalative binding to DNA.^16^ and complexes containing the dppn ligand exhibit strong intercalative binding interactions with duplex DNA (≥10^6^ M^–1^) via π-stacking with adjacent base pairs binding constants (≥10^6^ M^–1^).^15,17^ To date, a variety of DNA-binding dppn complexes have been reported including distorted square planar d^8^ platinum(II) complexes,^18^ a copper(II) complex,^19^ a half-sandwich rhodium(III) complex^20^ and acetate-bridged dirhodium(II) systems,^21^ as well as octahedral rhenium(I)^22^ osmium(II)^23^ and iridium(III) complexes.^24^ However, the most studied systems have been octahedral ruthenium(II) complexes. The first report of a dppn complex was by the Barton group in 1992, who studied the DNA binding of [Ru(phen)_2_(dppn)]^2+^, which was observed to exhibit weak luminescence enhancement when bound to dsDNA (∼25%).^25^ In addition, the quaternized dppn ligand has been shown to bind with good affinity to double stranded DNA (*K*_b_*ca* 10^6^ M^–1^) with quenching of emission by guanine-containing DNA and 4-fold luminescence enhancement observed in the presence of poly(dA*dT).^26^ Furthermore, DNA photocleavage has been observed for [Ru(bpy)_2_(dppn)]Cl_2_ through the low-lying ^3^ππ* state localized on the extended dppn ligand.^15,27−29^ The ligand-centered triplet excited state has been reported for several related Ru-dppn complexes^17,30−32^ and we recently reported the DNA binding and photophysics of the enantiomers of the [Ru(TAP)_2_(dppn)]Cl_2_ complex.^33^ Additionally, the 1,10-phenanthroline-containing *bis*-dppn complex [Ru(dppn)_2_(phen)]^2+^ has been shown to disrupt mitochondrial respiration^34^ and been investigated for potential photodynamic therapy of nonmelanoma skin cancer.^35^ In almost all cases, the activity toward DNA is attributed to the formation of singlet oxygen sensitized by the dppn-localized triplet state.

**Figure 1:**
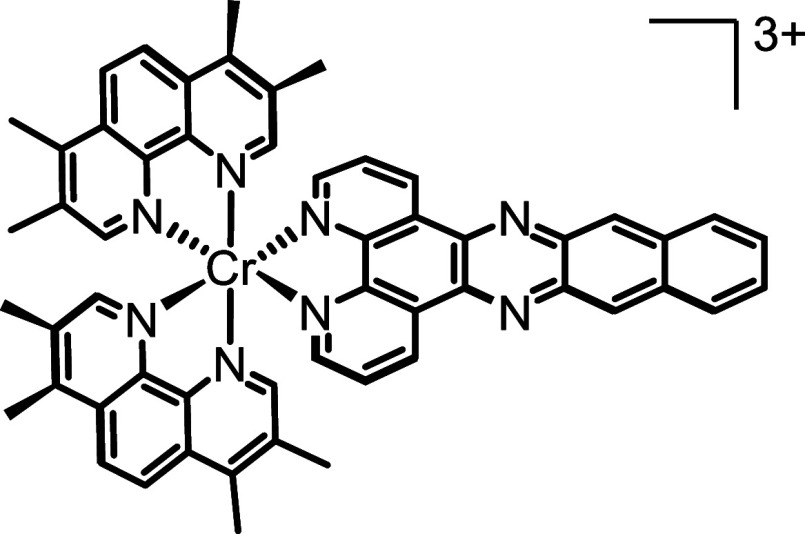
Structure of [Cr(TMP)_2_(dppn)]^3+^ (**1**).

Photoactive octahedral complexes of chromium(III) offer a sustainable alternative to ruthenium systems.^36−38^ The excited state photophysics of these d^3^-systems are now well-known and are dominated by the population of spin-flip metal-centered states of doublet multiplicity (^2^MC, e.g., ^2^E, ^2^T_1_).^38−41^ These long-lived metal-centered states are not only phosphorescent, but also strongly photo-oxidizing, being significantly more potent than their corresponding Ru(II)-polypyridyl analogues. For example, the ^2^E state of the archetypal [Cr(bpy)_3_]^3+^ has an excited state reduction potential of +1.44 V (vs NHE)^42^ in contrast to +0.84 V offered by the ^3^MLCT state of [Ru(bpy)_3_]^2+^.^43,44^ These Cr(III) complexes therefore typically surpass the electrochemical threshold for direct photo-oxidation of guanine bases. While prior focus has understandably been concerned with these favorable photoactive ^2^MC states,^44−50^ comparatively little attention has been paid to the role played by photo-oxidizing ligand-centered states within polypridyl complexes of Cr(III). We have previously explored the DNA binding behavior of chromium polypyridyl complexes containing the π-extended dipyrido[3,2-a:2′,3′-*c*]phenazine (dppz)^9,45−47^ and recently revealed the role of a short-lived ^1^LC dppz excited state ([^4^Cr(TMP)_2_(^1^dppz)]^3+^) rather than the ^2^MC state in the oxidation of DNA, which was only possible due to the close proximity of the intercalated ligand to the adenine base.^9^ Therefore, exploiting ligand-based excited states offers the potential to further extend the utility of photoactive chromium(III)-centered complexes as DNA-targeting agents. With this in mind, this study explores the photophysics and DNA binding of the dppn-containing Cr(III) complex [Cr(TMP)_2_(dppn)]^3+^ (**1**) for the first time, see Figure 1. The more bulky 3,4,7,8-tetramethyl-1,10-phenanthroline (TMP) ligand was chosen as a means to access complexes which are stable to racemization which would allow the study of the DNA binding of the resolved Λ and Δ enantiomers.^9,44^ Despite the number of dppn-containing complexes reported, there are very few studies that consider the DNA binding of chirally resolved dppn complexes.^51^ Interestingly, Barton and co-workers have shown that the presence of the bulky ligands leads to a preference for mis-match DNA^52^ and dppz complexes containing TMP ligands have shown a preference for AT DNA.^44,48,53,54^

## Results and Discussion

### Synthesis and Chiral Resolution

The heteroleptic complex *rac*-[Cr(TMP)_2_(dppn)]^3+^ (**1**) (TMP = 3,4,7,8-tetramethyl-1,10-phenanthroline; dppn = benzo[*i*]dipyrido[3,2-a:2′,3′-*c*]phenazine, Figure 1) was prepared through reaction of the triflate-containing precursor [Cr(TMP)_2_(OTf)_2_][OTf] with excess dppn in refluxing acetonitrile. Purification by size-exclusion chromatography followed by counterion metathesis with ^*n*^Bu_4_NPF_6_ yielded the hexafluorophosphate salt of **1** as an orange-colored solid with a modest yield of 48%. The identity of **1** was confirmed through mass spectrometry and elemental microanalysis, with a magnetic susceptibility of 3.91 μB as determined by Evans’ method being in good agreement with that expected for a d^3^-coordination complex with a quartet ground state electronic configuration (3.87 μB). Further counterion metathesis afforded the chloride salt of **1**, which not only displayed excellent aqueous solubility but also appeared stable to hydrolysis and ligand-exchange reactions when aqueous solutions were monitored over a 22-h period by electronic absorption spectroscopy (Figure S1). The Δ and Λ enantiomers of **1** were resolved by passing through a C25 Sephadex column eluted with an aqueous solution of (−)-O,O′-dibenzoyl-L-tartrate (Figure S2), yielding two yellow-colored solutions which displayed near-identical electronic absorption spectra (Figure S3). In previous unpublished measurements enantiopure solutions of the related [Cr(bpy)_2_(dppz)]^3+^ complex were found to racemize during the multiscan accumulation required to record a CD spectrum. For **1**, the retention of strong CD signals during multiple scanning cycles (Figure S4) confirms robustness to light-activated racemization under the illumination conditions required for spectroscopic measurement.

### Electrochemical Studies

An acetonitrile solution of **1** as its hexafluorophosphate salt was analyzed by cyclic voltammetry (Figures S5–S6 and Table S1) revealing an irreversible oxidation process at +1.31 V (vs Fc^+^/Fc) and a set of five closely spaced reduction waves between −0.50 V and the limit of the electrochemical solvent window at −2.70 V. The first, and fully electrochemically reversible reduction centered at −0.72 V is assigned to a dppn-based process, being anodically shifted relative to the first reduction process reported for both [Cr(TMP)_3_]^3+^ and [Cr(TMP)_2_(phen)]^3+^ in aqueous solution (−1.09 and −1.00 V respectively vs Fc^+^/Fc)^50^ and not dissimilar to the couple observed at ca. −0.80 V for [Cr(phen)_2_(dppz)]^3+^.^44,45,50^ The remaining reductive electrochemistry of **1** is complex, making definitive assignment challenging, although these processes likely involve reduction of the TMP moieties and further processes localized on the π-extended dppn ligand. The appearance of an oxidative wave at anodic potential is noteworthy as no oxidation processes have been previously reported for Cr(III) diimine systems featuring dppz or its substituted analogues.^44,45,56^ With the Cr(III/IV) couple being typically considered thermodynamically inaccessible,^57^ the wave at +1.31 V for **1** is assigned to a dppn-based oxidation. The increased π-system offered by the dppn ligand likely leads to a reordering of the frontier molecular orbitals in **1** compared to its dppz-counterpart and consequently results in a dppn-based HOMO. Indeed, a computational investigation of dppn and related π-extended ligand systems within complexes of Fe(II) reveals π-conjugation to have a stabilizing influence upon unoccupied π*-orbitals and, in some instances, a significant destabilizing effect upon ligand-based occupied orbitals of π-character.^58^ In summary, it is likely that both the HOMO and LUMO of **1** are dppn-based.

### Photophysical Studies

**Figure 2:**
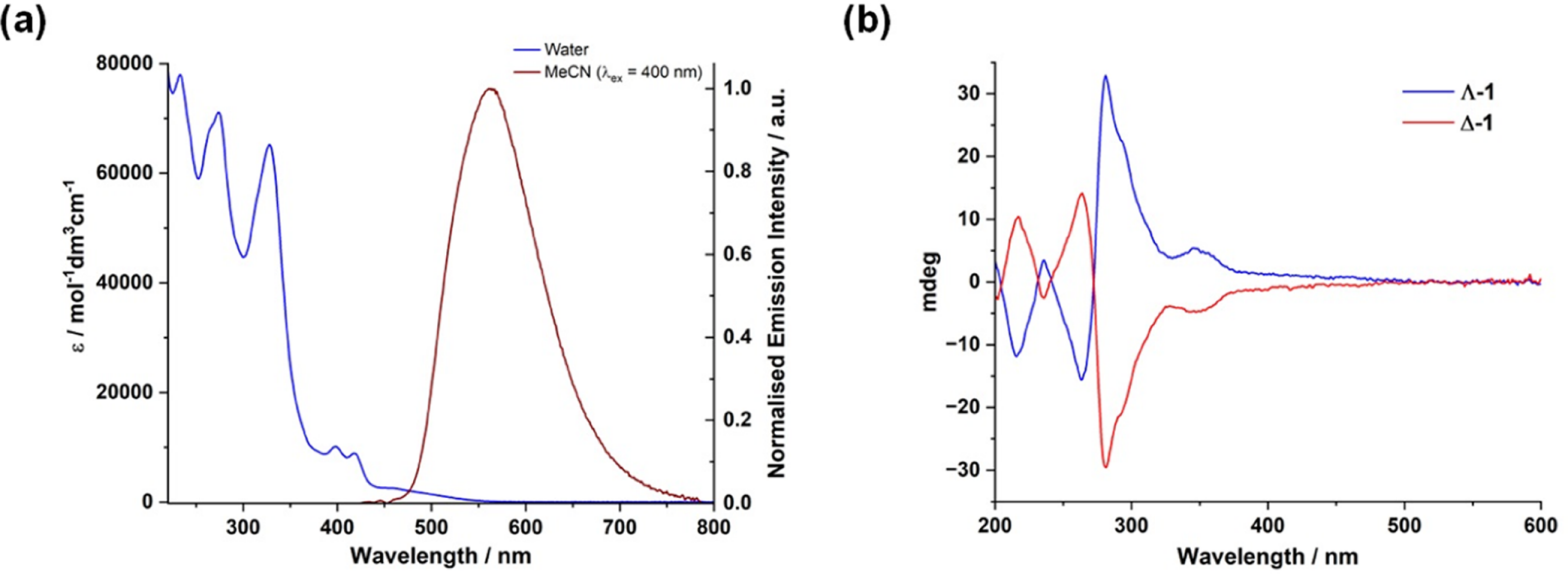
(a) UV–visible electronic absorption spectrum (blue) and normalized photoluminescence spectrum (red, λ_ex_ = 400 nm) recorded for aerated aqueous and MeCN solutions of **1** respectively. (b) Circular dichroism (CD) spectra recorded for aqueous solutions of **Λ-1** and **Δ-1** at room temperature.

The UV–visible electronic absorption spectrum of **1** in aqueous solution is shown in Figure 2. The UV region of the spectrum features several intense absorbances ascribed to ligand-localized π–π* transitions, with that centered at 330 nm likely involving the dppn moiety owing to its absence in the spectrum recorded for [Cr(TMP)_2_(dppz)]^3+^.^9,44^ Further, less intense, bands observed at 400 and 420 nm are seemingly characteristic of additional dppn-localized transitions owing to their appearance within the spectrum of the free ligand (Figure S7) and also for other transition metal complexes featuring this moiety.^14,17,22,24,25,59,60^ The broad, low-intensity tail encroaching into the visible region likely arises due to an admixture of ligand-to-metal charge transfer (LMCT) and ligand-centered excitations, obscuring Laporte forbidden ligand-field (d-d) transitions which are typically extremely weak (ε < 100 M^–1^dm^3^). Circular-dichroism (CD) spectra reveal equal and opposite features (Figure 2b), with the first enantiomer to elute displaying strong positive bands at 282 and 348 nm. The positive sign observed for the longer wavelength peaks in the π → π* region is assigned to the Λ-**1** enantiomer, in line with the application of exciton theory to similar complexes.^9,55,61^

While **1** is nonemissive in aqueous solution, excitation of an aerated acetonitrile solution at 400 nm results in a broad luminescence band at 563 nm with a lifetime of 30 ns (Φ_em_ = 1.0%) (Figures 2 and S8). The origin of this photoluminescence is ascribed to a dppn-localized excited state, with the profile aligning closely with that obtained following excitation of solutions of the free ligand (Figure S7) and resembling the luminescence behavior reported for other complexes featuring coordinated dppn.^17,24^ Luminescence was also observed for **1** in a frozen solvent glass at 77 K (Figure S8), being rigidochromically shifted to higher energy and exhibiting clear vibronic progressions at 503, 541, and 580 nm, consistent with assignment to a ligand-based emissive state. Interestingly, classical Cr-centered spin-flip luminescence from metal-centered excited states of doublet multiplicity (e.g., ^2^E and ^2^T_1_) was not observed for **1** under any of our experimental conditions, despite the ligand field strength of the employed donors likely being sufficient to favor their population over ^4^T_2_ levels. This suggests that excitation energy in **1** is efficiently funnelled to low-lying dppn-based excited states which are incapable of sensitizing the lower-lying metal-centered states.

### ps-Transient Absorption Spectroscopy

**Figure 3:**
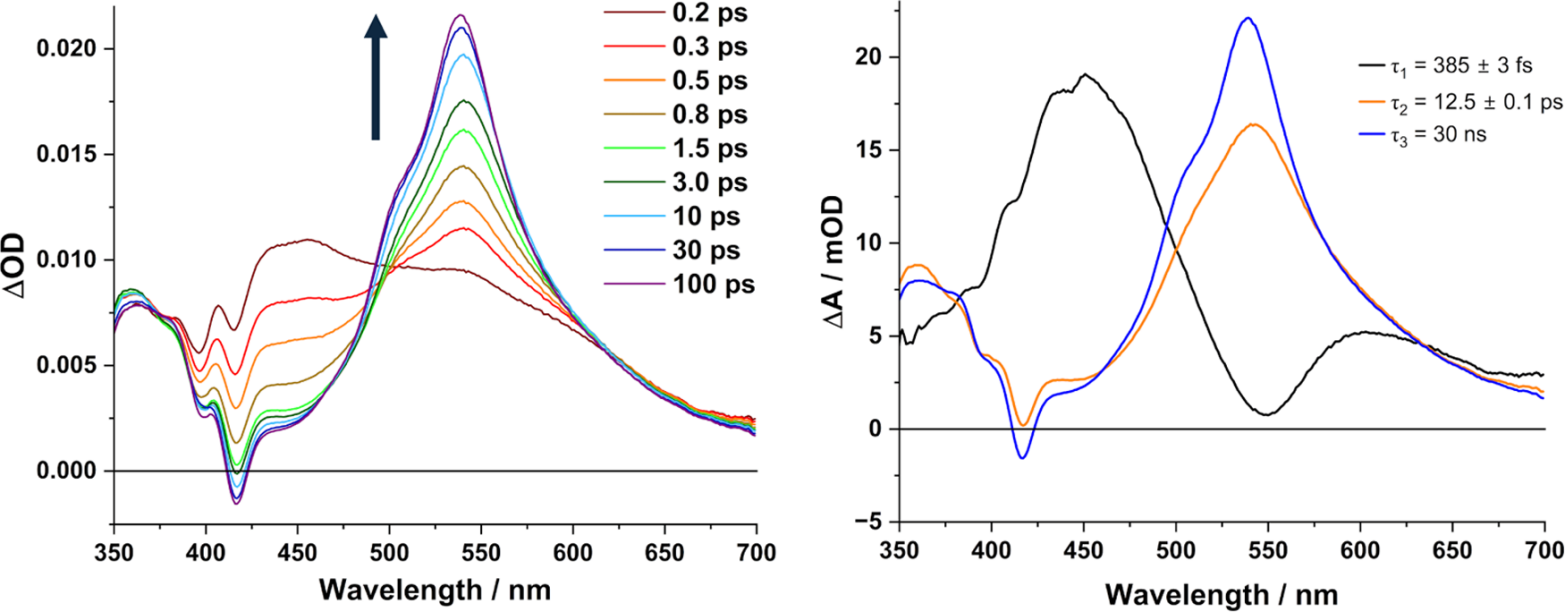
Left: Transient absorption spectra recorded for an aerated MeCN solution of **1** (λ_ex_ = 400 nm) showing detail of transients recorded between 0.2–100 ps following excitation. Right: Species associated spectra (SAS) extracted from global kinetic analysis.

The electronic excited state and associated dynamics of **1** were probed on the ultrafast time scale by transient absorption spectroscopy (Figure 3). Following excitation of an aerated MeCN solution of **1** with a 400 nm, 40 fs laser pulse, transient absorption spectra reveal the appearance of two discrete, broad bands. At early times, an excited state absorbance (ESA) feature is present centered around 450 nm, which kinetic analysis reveals to have an associated subpicosecond lifetime. Given the intensity and broad nature of this band, this initially captured excited state is tentatively assigned to a ligand-to-metal charge transfer (LMCT) state. Distinct negative-going signals in the ESA at 395 and 415 nm, together with a much broader feature between 450–550 nm all correspond to bleaching of the ground state, and indeed are present at all later times. After ca. 5 ps, the transient spectra evolve to be now dominated by a broad, intense band centered at 540 nm which persists beyond the time scale of the experiment (7 ns), with kinetic analysis suggesting a lifetime of 30 ns, identical to that determined by time-correlated single photon counting for the luminescence observed from **1** (*vide supra*). This latter ESA is near-identical to that observed on the ns time scale for previously reported Ru(II) dppn-containing complexes and is therefore straightforwardly assigned to an excited state absorption from the dppn-localized intraligand excited state.^15^ Global lifetime analysis reveals an additional component on the order of 10 ps (see SAS 2, Figure 3) associated with the initial evolution of this dppn-localized feature, being characterized by a slight shift to higher energy of all features, with the shoulder at 505 nm becoming more pronounced along with a deepening of ground state bleach features. We assign this component (τ_2_) as vibrational cooling of the ligand-localized excited state following population from the aforementioned short-lived charge transfer state and intersystem crossing.

### Singlet Oxygen Sensitization

With prior studies indicating that photoexcited dppn-containing transition metal complexes are effective sensitizers of singlet oxygen,^15^ we proceeded to determine the quantum yield of singlet oxygen sensitization for **1** in air-equilibrated MeCN solution. Direct measurement of O_2_(^1^Δ_g_) → ^3^O_2_ phosphorescence (λ_em_ = 1270 nm) gave Φ^1^O_2_ = 43%. With TrA studies (*vide supra*) revealing population of a long-lived dppn ^3^IL state for **1**, the yield of ^1^O_2_ suggests that access to this ligand-centered excited state allows particularly effective sensitization. Although this value is lower than that determined for the comparable Ru(II) system [Ru(phen)_2_(dppn)]^2+^ (Φ^1^O_2_ = 83%),^15^**1** is somewhat more effective than several other Ru(II) polypyridyl complexes for example, including the archetypal [Ru(bpy)_2_(dppz)]^2+^.^62−65^ Indeed, Φ^1^O_2_ for **1** is comparable to other Cr(III) systems where effective sensitization arises due to population of long-lived ^2^MC excited states,^66−68^ highlighting the capabilities of ligand-centered excited states within these systems in the effective generation of singlet oxygen and the potential role that they may play within type II PDT processes.

### DNA Spectroscopic Titrations

**Figure 4:**
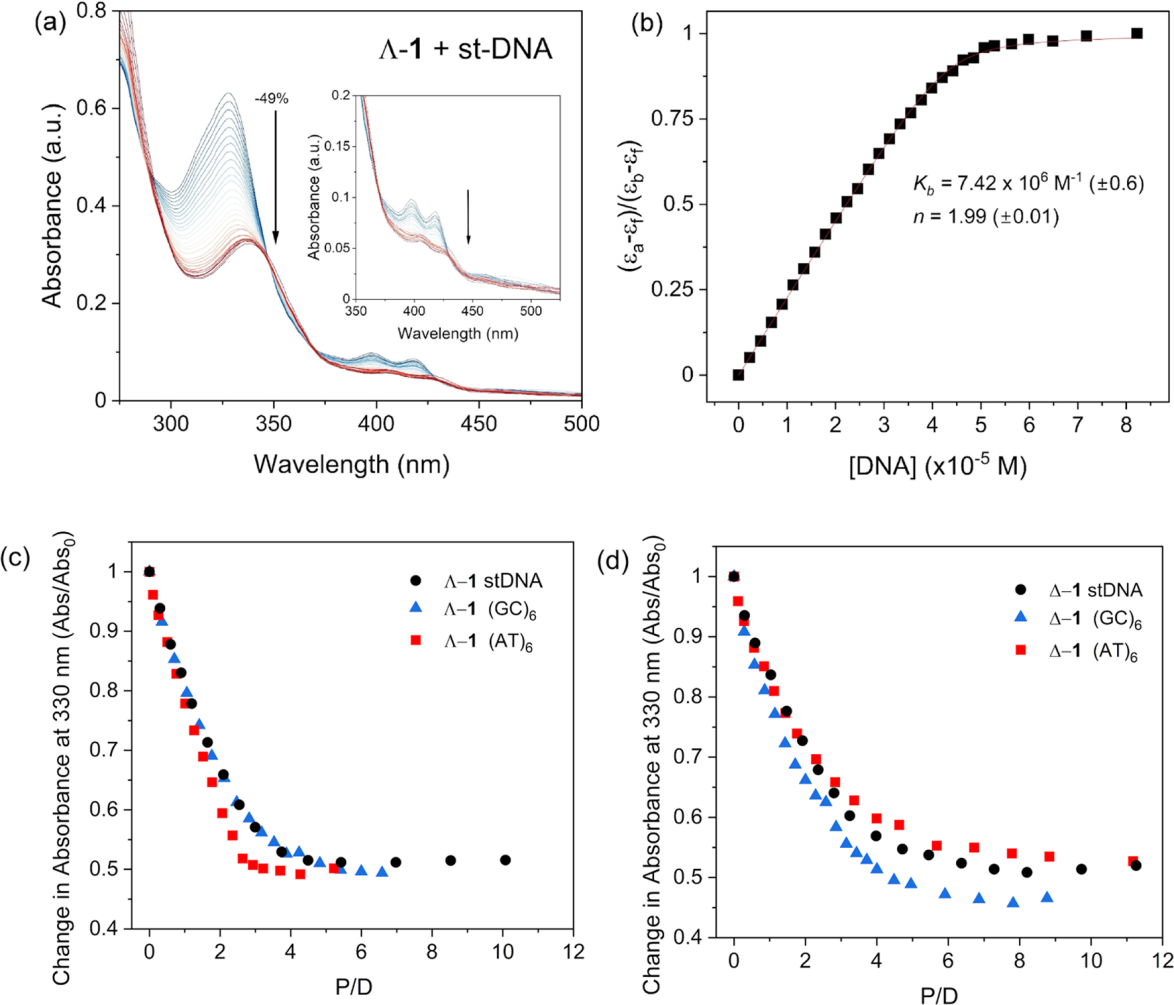
(a) DNA-binding titrations for Λ-**1** (10.9 μM) to ST-DNA (0–90 μM) in 50 mM phosphate buffer, pH 7. (b) Determination of the binding constant for Λ-**1** to ST-DNA determined at 330 nm vs [DNA] (per nucleobase) and nonlinear curve fitting of the data (**—**) using the method of Bard et al.^69^ (c) and (d) Comparative data of Abs/Abs_0_ at 330 nm of each enantiomer of [Cr(TMP)_2_(dppn)].3Cl vs the three different DNA systems.

Titrations of the Δ-**1** and Λ-**1** enantiomers with salmon testes natural DNA (st-DNA), which has a 32% GC content, were performed and monitored by UV–visible absorption spectroscopy. For both enantiomers a dramatic decrease in the absorbance (hypochromism) of the ligand-localized transitions at 330, 400 and 420 nm were observed, see Figure 4 and S9. The hypochromism measured at the most intense 330 nm band was found to be slightly greater for Λ-**1** (49%) compared to Δ-**1** (46%). These changes in absorbance intensity were accompanied by a shift in absorbance to longer wavelength (Δ-**1** 9 nm and Λ-**1** 11 nm) with well-defined isosbestic points observed (Figure S9). Such behavior is consistent with intercalation of the dppn ligand.^17^ Binding constants (*K*_b_) on the order of 10^6^ M^–1^ were derived from the absorption data using the Bard equation^69^ and revealed a slightly greater DNA binding affinity for the lambda enantiomer, see Table 1. The binding constants are slightly higher than those previously reported (*K*_b_ of 10^4^ to 10^5^ M^–1^) for the binding of dppz-containing chromium polypyridyl complexes ([Cr(N^∧^N)_2_(dppz)]^3+^) containing phen, bpy and TMP N^∧^N ancillary ligands to calf thymus DNA.^44^ The preference for the lambda enantiomer was previously observed for [Cr(phen)_2_(dppz)]^3+^ and the more closely related [Cr(TMP)_2_(dppz)]^3+^ for st-DNA.^9,44,50^ The spectroscopic changes observed are comparable to those seen for the binding of [Ru(bpy)_2_(dppn)]^2+^ to natural DNA where a *K*_b_*=* 2.2 × 10^6^ M^–1^ was observed with 37% hypochromism at 320 nm and a 10 nm redshift.^70^ The affinity is also comparable to that observed for the quaternized dppn ligand (*K*_b_*=* 1.33 × 10^6^ M^–1^ and *n* = 3).^26^ This is interesting as the TMP ligand would be expected to introduce more steric effects than present in the bipyridine-containing system and the free quaternized ligand.

**Table 1:** Summary of the Spectroscopic Changes and Binding Constant (*K*_b_) for the Enantiomers of **1** for Each DNA System^a^

DNA system	enantiomer	Δλ_max_/nm	(% hypochromism)	*K*_b_ (×10^6^ M^–1^)
st-DNA	Δ	+9 nm	–46%	2.64 (±0.44)
	Λ	+11 nm	–49%	5.47 (±0.80)
(AT)_6_	Δ	+8 nm	–51%	1.13 (±0.24)
	Λ	+11 nm	–46%	11.8 (±0.35)
(GC)_6_	Δ	+9 nm	–51%	1.17 (±0.53)
	Λ	+12 nm	–47%	2.39 (±0.32)
Htel(K)	Δ	+8 nm	–45%	0.62 (±0.12)
	Λ	+9 nm	–41%	1.84 (±0.37)

a Quoted values are the average of results from two titrations.

As the closely related [Cr(TMP)_2_(dppz)]^3+^ complex shows preference for AT DNA^9,44^ comparative DNA titrations were performed with double-stranded DNA formed by the d(AT)_6_ and d(GC)_6_ self-complementary dodecamer sequences, see Figures S10 and S11. A summary of the titration results is given in Table 1. Δ-**1** and Λ-**1** were found to bind to both d(AT)_6_ and d(GC)_6_ with good affinity with a characteristic shift in the absorbance accompanied by band hypochromism. Λ-**1** was found to bind to all three DNA systems with greater affinity than Δ-**1**, and of the three systems, Λ-**1** was observed to bind with greater affinity for AT-containing DNA following the trend (AT)_6_ > st-DNA > (GC)_6_. The order observed likely reflects the 68% AT content in st-DNA. The preference for AT DNA may arise due to either the dppn or the TMP ligand. For example, a Ru(II) complex containing dppn and trispyrazolylmethyl ligands exhibits greater affinity for AT-DNA compared to GC-DNA, which was attributed to the ability of AT tracts to accommodate the steric demand of ligands better than the deeper, more rigid GC tracts.^17^ Additionally, the dppn-containing iridium complex [Ir(ppy)_2_(dppn)]^3+^ (H-ppy = 2-Phenylpyridine) has been shown to bind poly(dA*dT) with a greater affinity than poly(dG*dC).^24^ The impact of ancillary ligands in [Cr(diimine)_2_(dppz)]^3+^ complexes on DNA binding has previously been described in the work by Vandiver et al.^44^ In that work equilibrium dialysis measurements confirmed a preference of [Cr(TMP)_2_(dppz)]^3+^ for AT DNA, which is attributed to binding in the minor groove. Due to the similar preference for AT DNA exhibited for complex **1**, we speculate that similar binding is occurring in this system. Interestingly, while **1** is found to be weakly luminescent in acetonitrile, no luminescence was observed under aqueous conditions where the Δ-**1** and Λ-**1** enantiomers were fully bound to any of the DNA systems, see Figure S13. This suggests that the binding site does not fully protect the dppn ligand from the solvent interactions. This could be explained by dppn binding from the minor groove and protruding into the major groove, which has been proposed by others.^25^ Finally, we considered the ability of Δ-**1** and Λ-**1** to bind to quadruplex DNA as we were interested to see if the extended dppn ligand would show good affinity for the Hoogsteen bonded guanine tetrad structure. To do this, titrations were performed with the potassium stabilized G4-quadruplex formed from the human telomer sequence hTel(K), see Figure S12. Interestingly, the lambda enantiomer was found to bind with greater affinity, though overall the complexes were found to bind more weakly to the quadruplex DNA.

### Thermal Denaturation Studies

**Figure 5:**
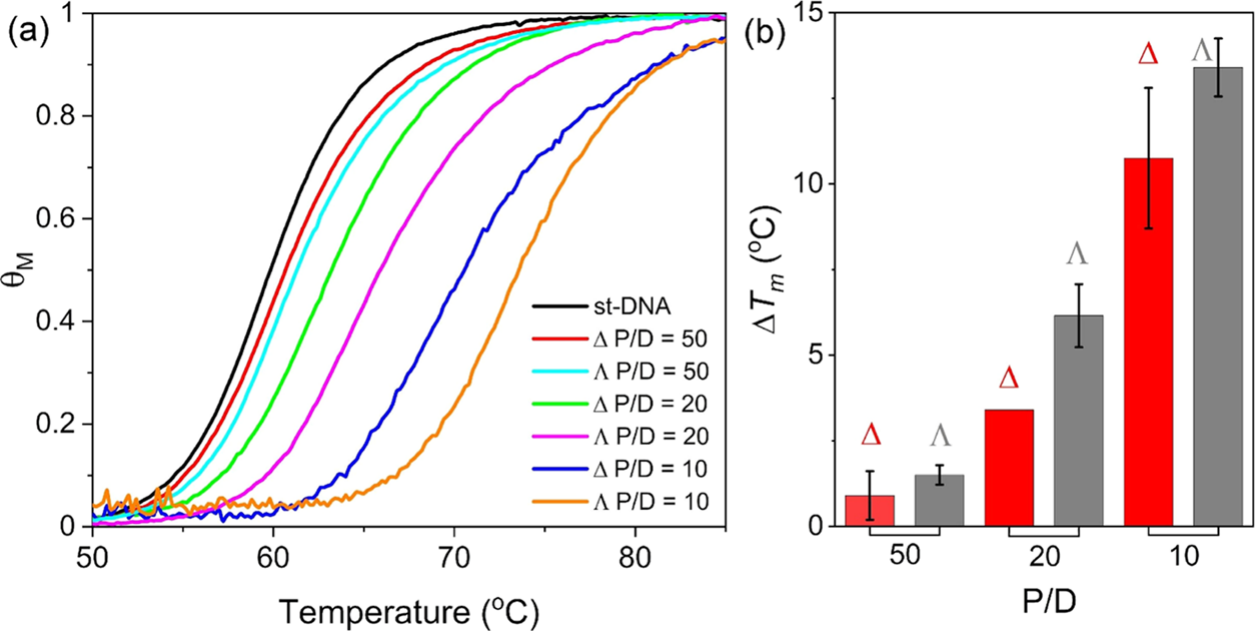
(a) Thermal denaturation results for Δ-**1** and Λ-**1** in the presence of st-DNA at P/D [DNA]/[Cr] ratios of 50, 20, and 10 all in 1 mM potassium phosphate buffer, 2 mM NaCl. (b) Difference in melting temperature (Δ*T*_m_) for Δ-**1** and Λ-**1** at different P/D ratios relative to st-DNA.

The DNA binding interactions of Δ-**1** and Λ-**1** were further studied by performing DNA denaturation experiments to determine the impact of binding on the stabilization of the double-stranded structure to heating. This was done by monitoring the absorbance at 260 nm of DNA solutions heated from 25 to 95 °C at nucleobase to complex (P/D) ratios of 10, 20, and 50. The melting temperature, *T*_m_, at which DNA is 50% denatured, was found to increase in the presence of both Δ-**1** and Λ-**1** see Figure 5, which supports stabilization via intercalation.^71^ Notably, at each P/D the Λ enantiomer shows greater increases in *T*_m_ compared to the Δ enantiomer, which is more readily seen by comparing the derivative of the melting curve obtained for each enantiomer as shown in Figure S14. The magnitude of the stabilization is reflected in the Δ*T*_m_, which is the difference in the *T*_m_ value observed for st-DNA in the absence of **1** (59 °C) and in the presence of **1**. These values are shown in Figure 5b and Table 2. At a P/D of 50, the stabilizing effect of the Δ enantiomer was negligible (+0.9 °C), while the Λ enantiomer stabilized the DNA a further 1.5 °C. Under the conditions of highest equivalent of Λ-**1**, at a P/D of 10, a *T*_m_ of 73.2 ± 0.8 °C was determined. The results indicate that the Λ enantiomer of **1** stabilizes the st-DNA duplex to a greater extent and are in good agreement with the observation of higher binding affinity in the DNA titrations.

**Table 2:** Summary of the Thermal Denaturation Study for Enantiomers of **1** in the Presence of st-DNA^a^

enantiomer	P/D = 50 *T*_m_ (°C)	P/D = 50 Δ*T*_m_	P/D = 20 *T*_m_ (°C)	P/D = 20 Δ*T*_m_	P/D = 10 *T*_m_ (°C)	P/D = 10 Δ*T*_m_
Δ	60.7 (±0.7)	+0.9	63.2 (±0.1)	+3.4	70.5 (±2.1)	+10.7
Λ	61.3 (±0.3)	+1.5	65.9 (±0.9)	+6.1	73.2 (±0.8)	+13.4

a Δ*T*_m_ is the change in the *T*_m_ compared to that observed for st-DNA in the absence of complex.

Kane-Maguire et al. previously showed that the Λ enantiomers of [Cr(phen)_2_(dppz)]^3+^ and [Cr(TMP)_2_(dppz)]^3+^ bind more strongly to DNA than the corresponding Δ enantiomers, and that the presence of methyl groups on the ancillary ligands increases this binding preference.^44,50^ These denaturation studies show that the dppn-containing Cr(III) polypyridyl complexes also demonstrate preferential binding for AT sites in DNA, which is reflected in the binding constants extracted from the spectroscopic titrations. Vasudevan et al.^45^ showed significant stabilization of calf thymus natural DNA (CT-DNA) to thermal denaturation by the presence of *rac*-[Cr(phen)_2_(dppz)]^3+^, and that substitution at the intercalating ligand by more lipophilic groups (e.g., Me_2_dppz) resulted in higher binding constants to CT-DNA for complexes of the family [Cr(phen)_2_(X_2_dppz)]^3+^.^45^ This aligns with the high binding constants found here for [Cr(TMP)_2_(dppn)]^3+^, bearing a more lipophilic terminal benzo-substitution on the intercalating ligand vs dppz.

In summary, complex **1** shows very good affinity for DNA and the DNA binding results indicate that the preference for AT DNA observed for the related [Cr(TMP)_2_(dppz)]^3+^ complex is also observed in **1**. Previously reported Cr(III) polypyridyl complexes have shown phosphorescence arising from their long-lived metal-centered states and also have been shown to photo-oxidize purine bases.^9,44−50,55^ In contrast, the introduction of the dppn ligand in **1** is found to yield dramatically different photophysical properties that render it nonluminescent in aqueous solution. Instead of a luminescent MC state, the excited state behavior is governed by the population of the dppn ligand-localized triplet excited state (τ = 30 ns), which is observed to sensitize singlet oxygen formation with a yield of 43%, which is comparable to previously reported values for Cr(III) polypyridyl complexes.^72^ The combination of singlet oxygen generation and good binding affinity provides an additional pathway for targeting DNA.

## Conclusions

The development of photoactive metallodrugs is seen as a promising avenue to more targeted treatment of disease and is the focus of significant efforts^1,2^ with the versatility of transition metal polypyridyl complexes making them ideal candidates,^3^ while complexes featuring extended aromatic units are more readily internalized by live cells (without the need for a transporting agent), due to their increased lipophilicity.^29,31^ Earth-abundant chromium(III) is an attractive alternative to more precious metals, with its complexes offering photofunctional metal-centered as well as ligand-centered excited states. This study is the first report of a chromium(III) polypyridyl complex containing a dppn ligand, expanding the rich coordination chemistry^72^ of complexes of this transition metal ion and opening the way for the further investigation of related complexes within photosensitizing applications.

## Experimental Section

### Materials

The ligand dppn was prepared from 1,10-phenanthroline-5,6-dione and 2,3-diaminonaphthalene following an adapted literature procedure,^74^ while the Cr(III) precursor complex [Cr(TMP)_2_(OTf)_2_][OTf] was synthesized following a known procedure utilizing 3,4,7,8,-tetramethyl-1,10-phenanthroline (TMP) as the diimine.^50,75^ All other reagents were obtained from commercial sources and were of reagent grade quality. The oligonucleotides were synthesized, desalted and purified (by gel filtration) by Eurogentec (Liege, Belgium). Salmon testes natural DNA was purchased from Sigma-Aldrich. d(AT)_2_ (ε_260 nm_ = 133,300 M^–1^ cm^–1^, single-stranded), d(GC)_2_ (ε_260 nm_ = 101,100 M^–1^ cm^–1^, single-stranded), h(tel) (ε_260 nm_ = 244,300 M^–1^ cm^–1^, single-stranded) and Salmon testes DNA (ε_260 nm_ = 6600 M^–1^ cm^–1^, nucleobase) concentrations were determined spectrophotometrically.

### Instruments and Methods

#### Reagents, Synthesis and Characterization

Anhydrous MeCN was obtained by distillation from CaH_2_, being stored over freshly activated 4 Å molecular sieves under an atmosphere of dry N_2_. All synthetic manipulations performed under an atmosphere of N_2_ employed standard Schlenk line techniques. Size-exclusion chromatography was performed under gravity using a fritted column of 35 mm diameter and 1000 mm length packed with Sephadex LH-20 resin which had previously been allowed to swell in 3:1 (*v*/*v*) MeOH/MeCN solution overnight. NMR spectra were acquired on a Bruker Ascend 400 MHz spectrometer, with chemical shifts reported relative to the residual solvent signal (CDCl_3_: ^1^H δ 7.26, ^13^C δ 77.16). High-resolution mass spectra were recorded on an Agilent 1290 Infinity II instrument with a 6545 QTOF mass analyzer. Infrared spectra were recorded on a Shimadzu IRSpirit FTIR spectrometer fitted with a QATR-S ATR accessory. Elemental microanalysis was conducted at London Metropolitan University. Magnetic susceptibility measurements were determined following Evan’s method, utilizing a coaxial NMR tube containing complex **1** as its hexafluorophosphate salt in a solution of *d*_3_-MeCN (580 μL) and ^*t*^BuOH (20 μL).

#### Photophysical Analysis

UV–visible absorption spectra were recorded on an Agilent Cary-60 spectrometer and solution state luminescence spectra on a Horiba Fluoromax-4 spectrometer using quartz cuvettes of 1 cm path length. Luminescence lifetimes were determined by time-correlated single photon counting (TCSPC) on an Edinburgh Instruments mini-τ, equipped with a ps diode laser (404 nm, 56 ps).

#### ps-Transient Absorption Spectroscopy

Transient optical absorption measurements (TrA) were performed at the Lord Porter Laser Laboratory at the University of Sheffield using a Helios system (HE-VIS-NIR-3200, Ultrafast Systems). Solutions of the sample were prepared in 2 mm path length quartz cuvettes to an optical density of 0.3 OD at 400 nm. The sample was probed by a UV–visible supercontinuum (340–800 nm), generated by pumping a CaF_2_ crystal with 800 nm light (10 kHz, 40 fs fwhm), while the sample was pumped by a 400 nm pump beam (2 mW, 5 kHz, 40 fs fwhm). Pump and probe pulses were linearly polarized and had their polarization set to the magic angle (54.7°). 200 time points were measured in the 7 ns range with an exponential distribution, beginning with 20 fs steps at δt = 0. For each time point, data was collected for 1.5 s, for 5 cycles.

#### Singlet Oxygen Sensitization

^1^O_2_ was detected via measurement of the fingerprint emission band at 1275 nm. Samples were prepared in solutions of MeCN at an optical density of approximately 0.4 OD at 355 nm. Samples were photoexcited by frequency tripled output of a Q-switched Nd:YAG laser (LOTIS-II LS-1231M) at 355 nm, with an 8 ns pulse length. The emission was filtered by a 1277 nm bandpass filter and focused onto a InGaAs photodiode (J22D-M204-R03M-60-1.7, Judson Technologies). The output was then coupled to a current amplifier (DLPCA-200, FEMTO Messtechnik GmbH) and recorded by a digital oscilloscope (TDS 3032B Tektronix).

The quantum yield was determined by comparison of the amplitude of the emission signal, averaged between different laser powers over the linear response region, with the amplitude of a standard. A sample of perinaphthenone (Φ ≈ 1) was prepared in the same solvent to a similar optical density. Discrepancies between the optical densities of the two solutions were accounted for by normalizing the emission amplitude by the exact optical density.

#### Electrochemical Analysis

Cyclic voltammograms were collected for a 1.4 mmol dm^–3^ solution of complex **1** as its hexafluorophosphate salt in N_2_-saturated anhydrous MeCN. Analyte solutions contained 0.2 mol dm^–3 *n*^Bu_4_NPF_6_ as supporting electrolyte. Measurements were performed at room temperature under a stream of dry N_2_ using a glassy carbon working electrode, a Pt wire counter and a Ag/AgCl reference. The applied potential was controlled using a PalmSens EmStat3 potentiostat. When comparing against electrochemical processes reported in the literature, the Fc^+^/Fc couple is assumed to come at +0.4 V vs Ag/AgCl, and at +0.6 V vs NHE.

### Synthesis of Dipyridonaphthazine (dppn)

1,10-Phenanthroline-5,6-dione (0.77 g, 3.65 mmol) and 2,3-diaminonaphthalene (0.64 g, 4.05 mmol) were combined in EtOH (150 mL) and refluxed for 16 h. The resulting orange colored suspension was cooled to 50 °C, the solids collected by filtration while warm, and then washed with MeOH and Et_2_O. The solids were dried *in vacuo*, yielding the product as a brick-orange colored powder. Yield = 1.09 g, 90%. ^1^H NMR (CDCl_3_, 400 MHz): 7.58–7.64 (m, 2H), 7.76 (dd, *J* = 4.4, 7.9 Hz, 2H), 8.14–8.20 (m, 2H), 8.88 (s, 2H), 9.23 (dd, *J* = 1.7, 4.4 Hz, 2H), 9.58 (dd, *J* = 1.7, 8.1 Hz, 2H), ^13^C NMR (CDCl_3_, 101 MHz): 124.38, 127.23, 127.91, 127.97, 128.71, 134.03, 134.56, 138.94, 142.31, 149.01, 152.90. HRMS (ES): Calc’d for C_22_H_13_N_4_: *m*/*z* = 333.1135; found: *m*/*z* = 333.1151 (MH^+^).

### Synthesis of [Cr(TMP)_2_(dppn)][PF_6_]_3_ (**1**)

To an oven-dried flask under an atmosphere of N_2_ were added [Cr(TMP)_2_(OTf)_2_][OTf] (0.64 g, 6.60 mmol), dppn (0.33 g, 9.94 mmol) and anhydrous MeCN (50 mL). The solution was heated to reflux for 22 h, cooled to room temperature and then filtered through a Celite pad to remove a small quantity of yellow-colored solids. The deep-red colored filtrate was evaporated to dryness. The crude product was purified by size-exclusion column chromatography (Sephadex LH-20, 3:1 (v/v) MeOH:MeCN), which after two passes yielded a bright-red colored solution which was reduced to a minimum volume by rotary evaporation. Dropping this solution slowly into a methanolic solution of excess ^*n*^Bu_4_NPF_6_ yielded an orange precipitate, which was collected by filtration, washed with MeOH followed by Et_2_O and dried *in vacuo*. Yield = 0.41 g, 48%. HRMS (ES): Calc’d for C_54_H_44_N_8_P_2_F_12_Cr *m*/*z* = 1146.2370, found *m*/*z* = 1146.2390 (M-PF_6_)^+^; Calc’d for C_54_H_44_N_8_PF_6_Cr *m*/*z* = 1001.2730, found *m*/*z* = 1001.2743 (M-2PF_6_)^+^; Calc’d for C_54_H_44_N_8_PF_6_Cr *m*/*z* = 500.6362, found *m*/*z* = 500.6388 (M-2PF_6_)^2+^; Calc’d for C_54_H_44_N_8_Cr *m*/*z* = 285.4359, found *m*/*z* = 285.4395 (M-3PF_6_)^3+^. Anal. Calc’d for C_54_H_44_N_8_P_3_F_18_Cr (%): C 50.21, H 3.43, N 8.67, found (%): C 49.33,(a) H 3.20, N 8.20. IR (ATR): υ̅/cm^–1^ = 1622 (w), 1602 (w), 1537 (m), 1487 (w), 1421 (m), 1305 (w), 1272 (m), 1249 (m), 1144 (w), 1080 (w), 1051 (m), 1030 (m), 903 (w), 878 (w), 829 (vs), 754 (m), 727 (m) 718 (m), 636 (m), 555 (s), 523 (m), 490 (m), 433 (m), 417 (m).

### Counterion Metathesis

[Cr(TMP)_2_(dppn)][PF_6_]_3_ (0.32 g, 0.25 mmol) and Dowex 1 × 8 Cl-form ion-exchange resin (0.72 g) were added to MeOH (20 mL) and stirred at r.t. in the dark for 70 min. The resin was removed by filtration and the filtrate evaporated to dryness. To ensure purity, the product as its chloride salt was subject to size-exclusion chromatography (Sephadex LH-20, 3:1 (*v*/*v*) MeOH/MeCN), which after one pass and evaporation of the solvent yielded a dark red residue. The residue was redissolved in the minimum volume of MeOH and dropped into excess rapidly stirring Et_2_O, affording an orange precipitate which was collected by filtration and dried *in vacuo*. Yield = 0.16 g, 68%. HRMS (ES): Calc’d for C_54_H_44_N_8_ClCr *m*/*z* = 445.6386, found *m*/*z* = 445.6392 (M-2Cl)^2+^; Calc’d for C_54_H_44_N_8_Cr *m*/*z* = 285.4359, found *m*/*z* = 285.4369 (M-3Cl)^3+^.

### Titration and Thermal Denaturation Procedures

Interactions of the enantiomers of the [Cr(TMP)_2_(dppn)].3Cl complex with different DNA systems were evaluated by titration of aliquots of DNA into a complex solution (10 μM) and monitoring the spectroscopic response in UV–vis absorption. DNA stock solution concentrations were determined spectroscopically ε_st-DNA_ = 6600 M^–1^ cm^–1^ per nucleotide, (ε_(AT)6_ = 133,300 M^–1^ cm^–1^ per single strand, ε_(GC)6_ = 101,100 M^–1^ cm^–1^ per single strand). Titrations were carried out in aqueous solution containing potassium phosphate buffer (50 mM). Results expressed in P/D refer to the ratio of [DNA]/[Cr] in solution. Binding constants were extracted from the titration data using a nonlinear curve fitting according to the method outlined by Bard et al.^69^

Thermal denaturation studies were carried out using an Agilent 3500 Multicell absorption spectrometer. DNA stock solutions (80 μM, 20 mL) were prepared in aqueous buffer (1 mM potassium phosphate, 2 mM NaCl). The solution was heated from 25 °C – 95 °C at a ramp rate of 1 °C min^–1^, with measurements at 260 and 800 nm (to ensure a stable baseline) taken at 0.2 °C intervals. Data analysis was carried out with baseline fitting on the data according to the following
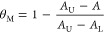
where θ_M_ is the fraction of denatured DNA in solution, *A* is the normalized absorbance, and *A*_U_ and *A*_L_ are the upper and lower fitted baselines, respectively. The temperature value which results in a θ_M_ value of 0.5 corresponds to the DNA melting temperature, *T*_m_. The measurements were carried out in the presence of increasing concentration ratios of [Cr(TMP)_2_(dppn)].3Cl to DNA (P/D 50, 20 and 10).

## Supporting Information Available

Additional experimental details, spectroscopic measurements and DNA binding titration data (PDF)

## Footnotes

(a) Elemental microanalysis suggests that complex **1** is isolated as its monohydrate: Anal. Calc’d for C_54_H_44_N_8_P_3_F_18_Cr.H_2_O: C 49.51, H 3.54, N 8.55, found (%): C 49.33, H 3.20, N 8.20.

## References

[ref1] Heinemann F.; Karges J.; Gasser G. Critical Overview of the Use of Ru(II) Polypyridyl Complexes as Photosensitizers in One-Photon and Two-Photon Photodynamic Therapy. Acc. Chem. Res. 2017, 50, 2727–2736. 10.1021/acs.accounts.7b00180. 29058879

[ref2] Monro S.; Colon K. L.; Yin H.; Roque J. 3rd; Konda P.; Gujar S.; Thummel R. P.; Lilge L.; Cameron C. G.; McFarland S. A. Transition Metal Complexes and Photodynamic Therapy from a Tumor-Centered Approach: Challenges, Opportunities, and Highlights from the Development of TLD1433. Chem. Rev. 2019, 119, 797–828. 10.1021/acs.chemrev.8b00211. 30295467 PMC6453754

[ref3] Shum J.; Leung P. K.-K.; Lo K. K.-W. Luminescent Ruthenium(II) Polypyridine Complexes for a Wide Variety of Biomolecular and Cellular Applications. Inorg. Chem. 2019, 58, 2231–2247. 10.1021/acs.inorgchem.8b02979. 30693762

[ref4] Burke C. S.; Byrne A.; Keyes T. E. Targeting Photoinduced DNA Destruction by Ru(II) Tetraazaphenanthrene in Live Cells by Signal Peptide. J. Am. Chem. Soc. 2018, 140, 6945–6955. 10.1021/jacs.8b02711. 29767962

[ref5] Poynton F. E.; Bright S. A.; Blasco S.; Williams D. C.; Kelly J. M.; Gunnlaugsson T. The development of ruthenium(ii) polypyridyl complexes and conjugates for in vitro cellular and in vivo applications. Chem. Soc. Rev. 2017, 46, 7706–7756. 10.1039/C7CS00680B. 29177281

[ref6] Knoll J. D.; Turro C. Control and utilization of ruthenium and rhodium metal complex excited states for photoactivated cancer therapy. Coord. Chem. Rev. 2015, 282–283, 110–126. 10.1016/j.ccr.2014.05.018. PMC4343038 25729089

[ref7] Moucheron C.; Kirsch-De Mesmaeker A.; Kelly J. M. Photoreactions of ruthenium (II) and osmium (II) complexes with deoxyribonucleic acid (DNA). J. Photochem. Photobiol. B: Biol. 1997, 40, 91–106. 10.1016/S1011-1344(97)00048-1. 9345780

[ref8] Keane P. M.; O’Sullivan K.; Poynton F. E.; Poulsen B. C.; Sazanovich I. V.; Towrie M.; Cardin C. J.; Sun X.-Z.; George M. W.; Gunnlaugsson T.; et al. Understanding the factors controlling the photo-oxidation of natural DNA by enantiomerically pure intercalating ruthenium polypyridyl complexes through TA/TRIR studies with polydeoxynucleotides and mixed sequence oligodeoxynucleotides. Chem. Sci. 2020, 11, 8600–8609. 10.1039/D0SC02413A. 34123120 PMC8163394

[ref9] Baptista F. A.; Krizsan D.; Stitch M.; Sazanovich I. V.; Clark I. P.; Towrie M.; Long C.; Martinez-Fernandez L.; Improta R.; Kane-Maguire N. A. P.; et al. Adenine Radical Cation Formation by a Ligand-Centered Excited State of an Intercalated Chromium Polypyridyl Complex Leads to Enhanced DNA Photo-oxidation. J. Am. Chem. Soc. 2021, 143, 14766–14779. 10.1021/jacs.1c06658. 34464120 PMC8447253

[ref10] Howerton B. S.; Heidary D. K.; Glazer E. C. Strained Ruthenium Complexes Are Potent Light-Activated Anticancer Agents. J. Am. Chem. Soc. 2012, 134, 8324–8327. 10.1021/ja3009677. 22553960

[ref11] Wachter E.; Howerton B. S.; Hall E. C.; Parkin S.; Glazer E. C. A new type of DNA ″light-switch″: a dual photochemical sensor and metalating agent for duplex and G-quadruplex DNA. Chem. Commun. 2014, 50, 311–313. 10.1039/C3CC47269H. 24226814

[ref12] Saxonov S.; Berg P.; Brutlag D. L. A genome-wide analysis of CpG dinucleotides in the human genome distinguishes two distinct classes of promoters. Proc. Natl. Acad. Sci. 2006, 103, 1412–1417. 10.1073/pnas.0510310103. 16432200 PMC1345710

[ref13] Chamberlain S.; Cole H. D.; Roque J. 3rd; Bellnier D.; McFarland S. A.; Shafirstein G. TLD1433-Mediated Photodynamic Therapy with an Optical Surface Applicator in the Treatment of Lung Cancer Cells In Vitro. Pharmaceuticals 2020, 13 (7), 137 10.3390/ph13070137. 32605213 PMC7407920

[ref14] Peña B.; Leed N. A.; Dunbar K. R.; Turro C. Excited State Dynamics of Two New Ru(II) Cyclometallated Dyes: Relation to Cells for Solar Energy Conversion and Comparison to Conventional Systems. J. Phys. Chem. C 2012, 116, 22186–22195. 10.1021/jp306352f.

[ref15] Foxon S. P.; Alamiry M. A. H.; Walker M. G.; Meijer A. J. H. M.; Sazanovich I. V.; Weinstein J. A.; Thomas J. A. Photophysical Properties and Singlet Oxygen Production by Ruthenium(II) Complexes of Benzo[i]dipyrido[3,2-a:2′,3′-c]phenazine: Spectroscopic and TD-DFT Study. J. Phys. Chem. A 2009, 113, 12754–12762. 10.1021/jp906716g. 19791785

[ref16] McConnell A. J.; Lim M. H.; Olmon E. D.; Song H.; Dervan E. E.; Barton J. K. Luminescent properties of ruthenium(II) complexes with sterically expansive ligands bound to DNA defects. Inorg. Chem. 2012, 51, 12511–12520. 10.1021/ic3019524. 23113594 PMC3622160

[ref17] Foxon S. P.; Metcalfe C.; Adams H.; Webb M.; Thomas J. A. Electrochemical and Photophysical Properties of DNA Metallo-intercalators Containing the Ruthenium(II) Tris(1-pyrazolyl)methane Unit. Inorg. Chem. 2007, 46, 409–416. 10.1021/ic0607134. 17279819

[ref18] Zamora A.; Wachter E.; Vera M.; Heidary D. K.; Rodríguez V.; Ortega E.; Fernández-Espín V.; Janiak C.; Glazer E. C.; Barone G.; Ruiz J. Organoplatinum(II) Complexes Self-Assemble and Recognize AT-Rich Duplex DNA Sequences. Inorg.Chem. 2021, 60, 2178–2187. 10.1021/acs.inorgchem.0c02648. 33502194 PMC8456496

[ref19] Barrett S.; De Franco M.; Kellett A.; Dempsey E.; Marzano C.; Erxleben A.; Gandin V.; Montagner D. Anticancer activity, DNA binding and cell mechanistic studies of estrogen-functionalised Cu(II) complexes. J. Biol. Inorg. Chem. 2020, 25, 49–60. 10.1007/s00775-019-01732-8. 31655896

[ref20] Scharwitz M. A.; Ott I.; Geldmacher Y.; Gust R.; Sheldrick W. S. Cytotoxic half-sandwich rhodium(III) complexes: Polypyridyl ligand influence on their DNA binding properties and cellular uptake. J. Organomet. Chem. 2008, 693, 2299–2309. 10.1016/j.jorganchem.2008.04.002.

[ref21] Joyce L. E.; Aguirre J. D.; Angeles-Boza A. M.; Chouai A.; Fu P. K. L.; Dunbar K. R.; Turro C. Photophysical Properties, DNA Photocleavage, and Photocytotoxicity of a Series of Dppn Dirhodium(II,II) Complexes. Inorg.Chem. 2010, 49, 5371–5376. 10.1021/ic100588d. 20496907

[ref22] Wing-Wah Yam V.; Kam-Wing Lo K.; Cheung K.-K.; Yuen-Chong Kong R. Deoxyribonucleic acid binding and photocleavage studies of rhenium(I) dipyridophenazine complexes. J. Chem. Soc., Dalton Trans. 1997, (12), 2067–2072. 10.1039/a700828g.

[ref23] Sun Y.; Joyce L. E.; Dickson N. M.; Turro C. DNA photocleavage by an osmium(II) complex in the PDT window. Chem. Commun. 2010, 46, 6759–6761. 10.1039/c0cc02571b. 20717583

[ref24] Lo K. K.; Chung C.-K.; Zhu N. Nucleic Acid Intercalators and Avidin Probes Derived from Luminescent Cyclometalated Iridium(III)–Dipyridoquinoxaline and – Dipyridophenazine Complexes. Chem.—Eur. J. 2006, 12, 1500–1512. 10.1002/chem.200500885. 16304644

[ref25] Hartshorn R. M.; Barton J. K. Novel dipyridophenazine complexes of ruthenium(II): exploring luminescent reporters of DNA. J. Am. Chem. Soc. 1992, 114, 5919–5925. 10.1021/ja00041a002.

[ref26] Phillips T.; Rajput C.; Twyman L.; Haq I.; Thomas J. A. Water-soluble organic dppz analogues—tuning DNA binding affinities, luminescence, and photo-redox properties. Chem. Commun. 2005, 34, 4327–4329. 10.1039/b506946g. 16113737

[ref27] Sun Y.; Joyce L. E.; Dickson N. M.; Turro C. Efficient DNA photocleavage by [Ru(bpy)2(dppn)]2+ with visible light. Chem. Commun. 2010, 46, 2426–2428. 10.1039/b925574e. 20379547

[ref28] Wang L.; Yin H.; Jabed M. A.; Hetu M.; Wang C.; Monro S.; Zhu X.; Kilina S.; McFarland S. A.; Sun W. pi-Expansive Heteroleptic Ruthenium(II) Complexes as Reverse Saturable Absorbers and Photosensitizers for Photodynamic Therapy. Inorg. Chem. 2017, 56, 3245–3259. 10.1021/acs.inorgchem.6b02624. 28263079

[ref29] Yin H.; Stephenson M.; Gibson J.; Sampson E.; Shi G.; Sainuddin T.; Monro S.; McFarland S. A. In Vitro Multiwavelength PDT with ^3^IL States: Teaching Old Molecules New Tricks. Inorg. Chem. 2014, 53, 4548–4559. 10.1021/ic5002368. 24725142

[ref30] Chen Y.; Lei W.; Jiang G.; Zhou Q.; Hou Y.; Li C.; Zhang B.; Wang X. A ruthenium(II) arene complex showing emission enhancement and photocleavage activity towards DNA from singlet and triplet excited states respectively. Dalton Trans. 2013, 42, 5924–5931. 10.1039/c3dt33090g. 23459918

[ref31] Albani B. A.; Peña B.; Leed N. A.; de Paula N. A.; Pavani C.; Baptista M. S.; Dunbar K. R.; Turro C. Marked improvement in photoinduced cell death by a new tris-heteroleptic complex with dual action: singlet oxygen sensitization and ligand dissociation. J. Am. Chem. Soc. 2014, 136, 17095–17101. 10.1021/ja508272h. 25393595

[ref32] Knoll J. D.; Albani B. A.; Turro C. Excited state investigation of a new Ru(ii) complex for dual reactivity with low energy light. Chem. Commun. 2015, 51 (42), 8777–8780. 10.1039/C5CC01865J. PMC4430371 25912170

[ref33] Stitch M.; Sanders R.; Sazanovich I. V.; Towrie M.; Botchway S. W.; Quinn S. J. Contrasting Photosensitized Processes of Ru(II) Polypyridyl Structural Isomers Containing Linear and Hooked Intercalating Ligands Bound to Guanine-Rich DNA. J. Phys. Chem. B 2024, 128, 7803–7812. 10.1021/acs.jpcb.4c04129. 39106822 PMC11331526

[ref34] Pozza M. D.; Mesdom P.; Abdullrahman A.; Prieto Otoya T. D.; Arnoux P.; Frochot C.; Niogret G.; Saubaméa B.; Burckel P.; Hall J. P.; et al. Increasing the π-Expansive Ligands in Ruthenium(II) Polypyridyl Complexes: Synthesis, Characterization, and Biological Evaluation for Photodynamic Therapy Applications. Inorg. Chem. 2023, 62, 18510–18523. 10.1021/acs.inorgchem.3c02606. 37913550

[ref35] Giacomazzo G. E.; Schlich M.; Casula L.; Galantini L.; Del Giudice A.; Pietraperzia G.; Sinico C.; Cencetti F.; Pecchioli S.; Valtancoli B.; et al. Ruthenium(ii) polypyridyl complexes with π-expansive ligands: synthesis and cubosome encapsulation for photodynamic therapy of non-melanoma skin cancer. Inorg. Chem. Front. 2023, 10, 3025–3036. 10.1039/D2QI02678C.

[ref36] Scattergood P. A. Recent advances in chromium coordination chemistry: luminescent materials and photocatalysis. Organomet. Chem. 2020, 43, 1–34. 10.1039/9781788017077-00001.

[ref37] Büldt L. A.; Wenger O. S. Chromium complexes for luminescence, solar cells, photoredox catalysis, upconversion, and phototriggered NO release. Chem. Sci. 2017, 8, 7359–7367. 10.1039/C7SC03372A. 29163886 PMC5672834

[ref38] Förster C.; Heinze K. Photophysics and photochemistry with Earth-abundant metals - fundamentals and concepts. Chem. Soc. Rev. 2020, 49 (4), 1057–1070. 10.1039/C9CS00573K. 32025671

[ref39] Kane-Maguire N. A. P. Photochemistry and Photophysics of Coordination Compounds: Chromium. In Topics in Current Chemistry; Balzani V.; Campagna S., Eds.; Springer: Berlin Heidelberg, 2007; pp 37–67.

[ref40] Kirk A. D. Photochemistry and Photophysics of Chromium(III) Complexes. Chem. Rev. 1999, 99, 1607–1640. 10.1021/cr960111+. 11849004

[ref41] Wagenknecht P. S.; Ford P. C. Metal centered ligand field excited states: Their roles in the design and performance of transition metal based photochemical molecular devices. Coord. Chem. Rev. 2011, 255, 591–616. 10.1016/j.ccr.2010.11.016.

[ref42] Brunschwig B.; Sutin N. Reactions of the excited states of substituted polypyridinechromium(III) complexes with oxygen, iron(II) ions, ruthenium(II) and -(III), and osmium(II) and -(III) complexes. J. Am. Chem. Soc. 1978, 100, 7568–7577. 10.1021/ja00492a023.

[ref43] Creutz C.; Sutin N. Electron-transfer reactions of excited states. Reductive quenching of the tris(2,2′-bipyridine)ruthenium(II) luminescence. Inorg. Chem. 1976, 15, 496–499. 10.1021/ic50156a062.

[ref44] Vandiver M. S.; Bridges E. P.; Koon R. L.; Kinnaird A. N.; Glaeser J. W.; Campbell J. F.; Priedemann C. J.; Rosenblatt W. T.; Herbert B. J.; Wheeler S. K.; et al. Effect of ancillary ligands on the DNA interaction of [Cr(diimine)_3_]^3+^ complexes containing the intercalating dipyridophenazine ligand. Inorg. Chem. 2010, 49, 839–848. 10.1021/ic9013619. 20039692

[ref45] Vasudevan S.; Smith J. A.; Wojdyla M.; McCabe T.; Fletcher N. C.; Quinn S. J.; Kelly J. M. Substituted dipyridophenazine complexes of Cr(III): Synthesis, enantiomeric resolution and binding interactions with calf thymus DNA. Dalton Trans. 2010, 39, 3990–3998. 10.1039/c000150c. 20372725

[ref46] Wojdyla M.; Smith J. A.; Vasudevan S.; Quinn S. J.; Kelly J. M. Excited state behaviour of substituted dipyridophenazine Cr(III) complexes in the presence of nucleic acids. Photochem. Photobiol. Sci. 2010, 9, 1196–1202. 10.1039/c0pp00110d. 20617266

[ref47] Devereux S. J.; Keane P. M.; Vasudevan S.; Sazanovich I. V.; Towrie M.; Cao Q.; Sun X.-Z.; George M. W.; Cardin C. J.; Kane-Maguire N. A. P.; et al. Study of picosecond processes of an intercalated dipyridophenazine Cr(III) complex bound to defined sequence DNAs using transient absorption and time-resolved infrared methods. Dalton Trans. 2014, 43, 17606–17609. 10.1039/C4DT01989J. 25182384

[ref48] Goforth S. K.; Gill T. W.; Weisbruch A. E.; Kane-Maguire K. A.; Helsel M. E.; Sun K. W.; Rodgers H. D.; Stanley F. E.; Goudy S. R.; Wheeler S. K.; et al. Synthesis of cis-[Cr(diimine)2(1-methylimidazole)2](3+) Complexes and an Investigation of Their Interaction with Mononucleotides and Polynucleotides. Inorg. Chem. 2016, 55 (4), 1516–1526. 10.1021/acs.inorgchem.5b02323. 26836266

[ref49] Kane-Maguire N. A. P.; Wheeler J. F. Photoredox behavior and chiral discrimination of DNA bound M(diimine)^3n+^ complexes (M = Ru^2+^, Cr^3+^). Coord. Chem. Rev. 2001, 211, 145–162. 10.1016/S0010-8545(00)00280-0.

[ref50] Barker K. D.; Barnett K. A.; Connell S. M.; Glaeser J. W.; Wallace A. J.; Wildsmith J.; Herbert B. J.; Wheeler J. F.; Kane-Maguire N. A. P. Synthesis and characterization of heteroleptic Cr(diimine)_3_^3+^ complexes. Inorg. Chim. Acta 2001, 316, 41–49. 10.1016/S0020-1693(01)00377-2.

[ref51] Choi S.-D.; Kim M.-S.; Kim S. K.; Lincoln P.; Tuite E.; Nordén B. Binding Mode of [Ruthenium(II) (1,10-Phenanthroline)_2_L]^2+^ with Poly(dT*dA-dT) Triplex. Ligand Size Effect on Third-Strand Stabilization. Biochemistry 1997, 36, 214–223. 10.1021/bi961675a. 8993336

[ref52] Boynton A. N.; Marcelis L.; Barton J. K. [Ru(Me4phen)_2_dppz](2+), a Light Switch for DNA Mismatches. J. Am. Chem. Soc. 2016, 138 (15), 5020–5023. 10.1021/jacs.6b02022. 27068529 PMC4989906

[ref53] Rajendiran V.; Palaniandavar M.; Periasamy V. S.; Akbarsha M. A. New [Ru(5,6-dmp/3,4,7,8-tmp)(2)(diimine)](2)(+) complexes: non-covalent DNA and protein binding, anticancer activity and fluorescent probes for nuclear and protein components. J. Inorg. Biochem. 2012, 116, 151–162. 10.1016/j.jinorgbio.2012.06.005. 23022692

[ref54] Das D.; Mondal P. Interaction of ruthenium(ii) antitumor complexes with d(ATATAT)_2_ and d(GCGCGC)_2_: a theoretical study. New J. Chem. 2015, 39, 2515–2522. 10.1039/C4NJ02118E.

[ref55] Barker K. D.; Benoit B. R.; Bordelon J. A.; Davis R. J.; Delmas A. S.; Mytykh O. V.; Petty J. T.; Wheeler J. F.; Kane-Maguire N. A. P. Intercalative binding and photoredox behavior of [Cr(phen)_2_(dppz)]^3+^ with B-DNA. Inorg. Chim. Acta 2001, 322, 74–78. 10.1016/S0020-1693(01)00555-2.

[ref56] Donnay E. G.; Schaeper J. P.; Brooksbank R. D.; Fox J. L.; Potts R. G.; Davidson R. M.; Wheeler J. F.; Kane-Maguire N. A. P. Synthesis and characterization of tris(heteroleptic) diimine complexes of chromium(III). Inorg. Chim. Acta 2007, 360, 3272–3280. 10.1016/j.ica.2007.03.055.

[ref57] Otto S.; Grabolle M.; Förster C.; Kreitner C.; Resch-Genger U.; Heinze K. [Cr(ddpd)_2_]^3+^: A Molecular, Water-Soluble, Highly NIR-Emissive Ruby Analogue. Angew. Chem., Int. Ed. 2015, 54, 11572–11576. 10.1002/anie.201504894. 26267153

[ref58] Curtin G. M.; Jakubikova E. Extended π-Conjugated Ligands Tune Excited-State Energies of Iron(II) Polypyridine Dyes. Inorg.Chem. 2022, 61, 18850–18860. 10.1021/acs.inorgchem.2c02362. 36367743

[ref59] Wang L.; Yin H.; Jabed M. A.; Hetu M.; Wang C.; Monro S.; Zhu X.; Kilina S.; McFarland S. A.; Sun W. π-Expansive Heteroleptic Ruthenium(II) Complexes as Reverse Saturable Absorbers and Photosensitizers for Photodynamic Therapy. Inorg.Chem. 2017, 56, 3245–3259. 10.1021/acs.inorgchem.6b02624. 28263079

[ref60] Huang J.-G.; Jin J.; Yuan Q.-Z.; Yang X.-X.; Cao D.-K.; Liu C. Benzo[g]quinoxaline-Based Complexes [Ir(pbt)_2_(dppn)]Cl and [Ir(pt)_2_(dppn)]Cl: Modulation of Photo-Oxidation Activity and Light-Controlled Luminescence. Inorg. Chem. 2023, 62 (26), 10382–10388. 10.1021/acs.inorgchem.3c01267. 37348470

[ref61] Mason S. F.; Peart B. J. Optical rotatory power of co-ordination compounds. Part XVII. The circular dichroism of trisbipyridyl and trisphenanthroline complexes. J. Chem. Soc., Dalton Trans. 1973, 949–955. 10.1039/dt9730000949.

[ref62] Hergueta-Bravo A.; Jiménez-Hernández M. E.; Montero F.; Oliveros E.; Orellana G. Singlet Oxygen-Mediated DNA Photocleavage with Ru(II) Polypyridyl Complexes. J. Phys.Chem. B 2002, 106, 4010–4017. 10.1021/jp013542r.

[ref63] Zhang S.-Q.; Meng T.-T.; Li J.; Hong F.; Liu J.; Wang Y.; Gao L.-H.; Zhao H.; Wang K.-Z. Near-IR/Visible-Emitting Thiophenyl-Based Ru(II) Complexes: Efficient Photodynamic Therapy, Cellular Uptake, and DNA Binding. Inorg. Chem. 2019, 58, 14244–14259. 10.1021/acs.inorgchem.9b02420. 31595752

[ref64] Sentagne C.; Chambron J.-C.; Sauvage J.-P.; Paillous N. Tuning the mechanism of DNA cleavage photosensitized by ruthenium dipyridophenazine complexes by varying the structure of the two non intercalating ligands. J. Photochem. Photobiol. B: Biol. 1994, 26, 165–174. 10.1016/1011-1344(94)07031-8. 7815190

[ref65] Oladipupo O. E.; Brown S. R.; Lamb R. W.; Gray J. L.; Cameron C. G.; DeRegnaucourt A. R.; Ward N. A.; Hall J. F.; Xu Y.; Petersen C. M.; et al. Light-responsive and Protic Ruthenium Compounds Bearing Bathophenanthroline and Dihydroxybipyridine Ligands Achieve Nanomolar Toxicity towards Breast Cancer Cells. Photochem. Photobiol. 2022, 98, 102–116. 10.1111/php.13508. 34411308 PMC8810589

[ref66] Jones R. W.; Auty A. J.; Wu G.; Persson P.; Appleby M. V.; Chekulaev D.; Rice C. R.; Weinstein J. A.; Elliott P. I. P.; Scattergood P. A. Direct Determination of the Rate of Intersystem Crossing in a Near-IR Luminescent Cr(III) Triazolyl Complex. J. Am. Chem. Soc. 2023, 145 (22), 12081–12092. 10.1021/jacs.3c01543. 37224437 PMC10251520

[ref67] Basu U.; Otto S.; Heinze K.; Gasser G. Biological Evaluation of the NIR-Emissive Ruby Analogue [Cr(ddpd)_2_][BF_4_]^3+^ as a Photodynamic Therapy Photosensitizer. Eur. J. Inorg. Chem. 2019, 2019, 37–41. 10.1002/ejic.201801023.

[ref68] Otto S.; Nauth A. M.; Ermilov E.; Scholz N.; Friedrich A.; Resch-Genger U.; Lochbrunner S.; Opatz T.; Heinze K. Photo-Chromium: Sensitizer for Visible-Light-Induced Oxidative C–H Bond Functionalization—Electron or Energy Transfer?. ChemPhotoChem 2017, 1, 344–349. 10.1002/cptc.201700077.

[ref69] Carter M. T.; Rodriguez M.; Bard A. J. Voltammetric studies of the interaction of metal chelates with DNA. 2. Tris-chelated complexes of cobalt(III) and iron(II) with 1,10-phenanthroline and 2,2′-bipyridine. J. Am. Chem. Soc. 1989, 111, 8901–8911. 10.1021/ja00206a020.

[ref70] Zhou Q.-X.; Lei W.-H.; Chen J.-R.; Li C.; Hou Y.-J.; Wang X.-S.; Zhang B.-W. A New Heteroleptic Ruthenium(II) Polypyridyl Complex with Long-Wavelength Absorption and High Singlet-Oxygen Quantum Yield. Chem.—Eur. J. 2010, 16, 3157–3165. 10.1002/chem.200902563. 20108277

[ref71] Kelly J. M.; Tossi A. B.; McConnell D. J.; OhUigin C. A study of the interactions of some polypyridylruthenium (II) complexes with DNA using fluorescence spectroscopy, topoisomerisation and thermal denaturation. Nucleic Acids Res. 1985, 13, 6017–6034. 10.1093/nar/13.17.6017. 4047939 PMC321935

[ref72] Alazaly A. M. M.; Clarkson G. J.; Ward M. D.; Abdel-Shafi A. A. Mechanism of Oxygen Quenching of the Excited States of Heteroleptic Chromium(III) Phenanthroline Derivatives. Inorg. Chem. 2023, 62, 16101–16113. 10.1021/acs.inorgchem.3c02343. 37721399 PMC10548418

[ref74] Yam V. W.-W.; Lo K. K.-W.; Cheung K.-K.; Kong R. Y.-C. Synthesis, photophysical properties and DNA binding studies of novel luminescent rhenium(I) complexes. X-Ray crystal structure of [Re(dppn)(CO)_3_(py)](OTf). J. Chem. Soc., Chem. Commun. 1995, 1191–1193. 10.1039/c39950001191.

[ref75] Ryu C. K.; Endicott J. F. Synthesis, spectroscopy, and photophysical behavior of mixed-ligand mono- and bis(polypyridyl)chromium(III) complexes. Examples of efficient, thermally activated excited-state relaxation without back intersystem crossing. Inorg. Chem. 1988, 27, 2203–2214. 10.1021/ic00286a002.

