# Supplementary material for: Study of the Photophysical Properties and the DNA Binding of Enantiopure [Cr(TMP)2(dppn)]3+ Complex
Source: Inorg Chem. 2024 Dec 3;63(50):23620–9. doi: 10.1021/acs.inorgchem.4c03590 (PMC11653251; doi:10.1021/acs.inorgchem.4c03590)
Supplement: Supplementary file 1 — ic4c03590_si_001.pdf [file ic4c03590_si_001.pdf]

Supporting Information

Study of the Photophysical Properties and the DNA Binding of Enantiopure $[Cr(TMP)_2(dppn)]^{3+}$ Complex

Daniel Graczyk,[1] Rory A. Cowin,[2] Dimitri Chekulaev,[2] Maisie A. Haigh,[3] Paul A. Scattergood,[3*] and Susan J. Quinn[1*]

[1]School of Chemistry, University College Dublin, Dublin 4, Ireland

[2]Department of Chemistry, University of Sheffield, Brook Hill, Sheffield, S1 3HF, UK

[3]Department of Chemistry, School of Applied Sciences, University of Huddersfield, Queensgate, Huddersfield HD1 3DH, UK

Instrumental Methods:

Circular dichroism measurements were recorded on a Jasco J-810 spectropolarimeter in a quartz cuvette with a path length of 1 cm. The CD measurements reported were averaged over 8 scans with manual baseline subtraction. UV-Vis absorption measurements were recorded during DNA titrations on a Varian Cary 50 spectrometer, using a quartz cuvette with a path length of 1 cm. Thermal denaturation experiments were carried out using an Agilent 3500 Multicell Peltier spectrometer, using quartz cuvettes fitted with internal temperature probes.

Chiral Resolution of $[Cr(TMP)_2(dppn)]^{3+}$:

CM-Sephadex C-25 cation exchanger was swelled overnight in deionised water and degassed under vacuum for 1 h. This stationary phase was loaded into a 1-metre-long column with a peristaltic pump connected in a recirculating configuration and allowed to settle overnight. $[Cr(TMP)_2(dppn)].3PF_6$ (10 mg) was converted to the water-soluble chloride salt by shaking in the presence of Dowex 1X4 Chloride form ion exchange resin for 1 h. in deionised water.

The solution of the racemic chloride salt was loaded onto the column, and deionised water was passed through the column until all the complex was adsorbed to the surface of the Sephadex, indicated by the water above being colourless. Sephadex was added on top of the loading bed to fill the volume of the column. Deionised water was passed through the column at a flow rate of 0.1 mL min^{-1} initially, gradually increasing to 1 mL min^{-1}, for 30 min. The mobile phase was changed to a solution of sodium (-)-O,O'-dibenzoyl-L-tartrate (0.1 M), upon which the band of loaded complex began to move through the column. The top column plunger was lowered after introduction of the tartrate mobile phase as the increased ionic strength resulted in shrinking of the Sephadex. As the complex reached the bottom of the column, the flow rate was gradually lowered to 0.1 mL min^{-1} to prevent mixing during recirculation. During the

resolution procedure, the column was kept in the dark. Separate bands were observed after 3 recirculations, and the bands collected. The tartrate salt of the complex was converted to the chloride by shaking in the presence of Dowex 1X4 chloride form, where the removal of tartrate was monitored by UV-Vis absorption spectroscopy.

The chloride salt was recovered by evaporation under vacuum and desalted by stirring in MeCN. The solution was filtered and evaporated, and deionised water added. The hexafluorophosphate salt of the complex was recovered by addition of excess solid NH_4PF_6. The precipitate was washed by centrifugation to remove excess NH_4PF_6, and the enantiomers were stored as a solid at 4 °C. Circular dichroism spectroscopy was used to determine successful resolution of the enantiomers. (Enantiomer assignment based on Baptista *et al.*, 2021)[1].

Titration & Thermal Denaturation Procedures:

Interactions of the enantiomers of the $[Cr(TMP)_2(dppn)].3Cl$ complex with different DNA systems were evaluated by titration of aliquots of DNA into a complex solution (10 μM) and monitoring the spectroscopic response in UV-Vis absorption. DNA stock solution concentrations were determined spectroscopically ($\varepsilon_{st\text{-}DNA}$ = 6600 M^{-1} cm^{-1} per nucleotide, ($\varepsilon_{(AT)6}$ = 133,300 M^{-1} cm^{-1} per single strand, $\varepsilon_{(GC)6}$ = 101,100 M^{-1} cm^{-1} per single strand). Titrations were carried out in aqueous solution containing potassium phosphate buffer (50 mM). Results expressed in P/D refer to the ratio of [DNA]:[Cr] in solution. Binding constants were extracted from the titration data using a non-linear curve fitting according to the method outlined by Bard *et al.*[2]

Thermal denaturation studies were carried out using an Agilent 3500 Multicell absorption spectrometer. DNA stock solutions (80 μM, 20 mL) were prepared in aqueous buffer (1 mM potassium phosphate, 2 mM NaCl). The solution was heated from 25 °C – 95 °C at a ramp rate of 1 °C min^{-1}, with measurements at 260 nm and 800 nm (to ensure a stable baseline) taken at 0.2 °C intervals. Data analysis was carried out with baseline fitting on the data according to the following:

$$\theta_M = 1 - \frac{A_U - A}{A_U - A_L}$$

, where θ_M is the fraction of denatured DNA in solution, A is the normalised absorbance, and A_U and A_L are the upper and lower fitted baselines respectively. The temperature value which results in a θ_M value of 0.5 corresponds to the DNA melting temperature T_m. The measurements were carried out in the presence of increasing concentration ratios of $[Cr(TMP)_2(dppn)].3Cl$ to DNA (P/D 50, 20 and 10).

Figures

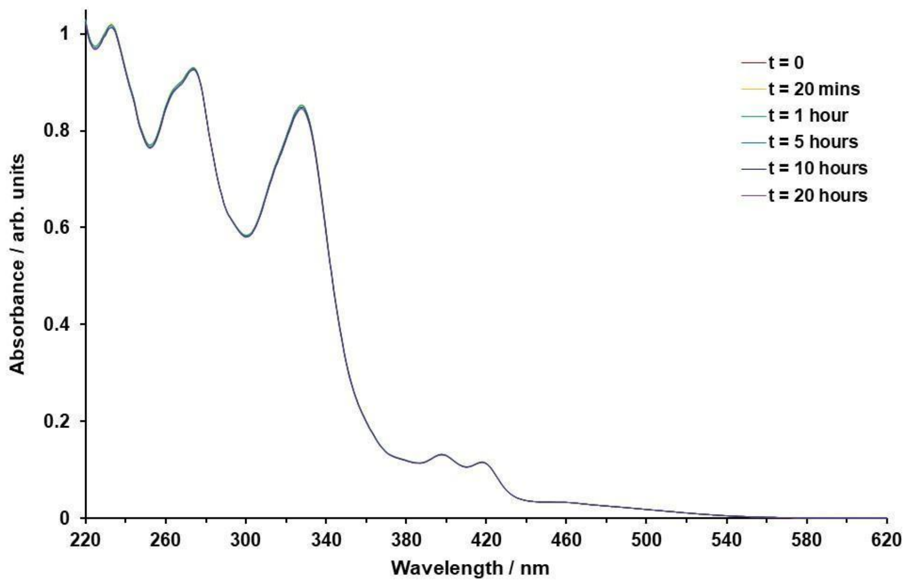

Figure S1 UV-Visible electronic absorption spectra recorded for an aqueous solution of *rac*-**1** stored in the dark over 20 hours.

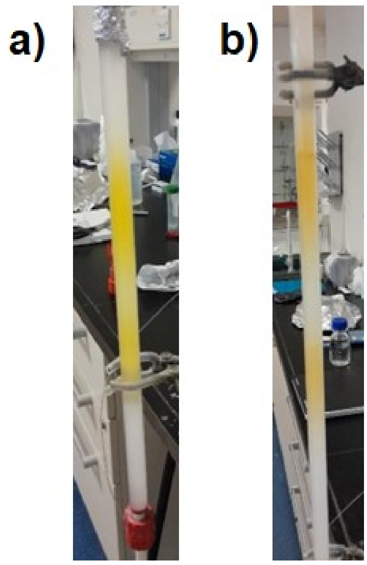

Figure S2 Complex **1** undergoing chiral-resolution by column chromatography, showing the two bands after the second (a) and third (b) passes.

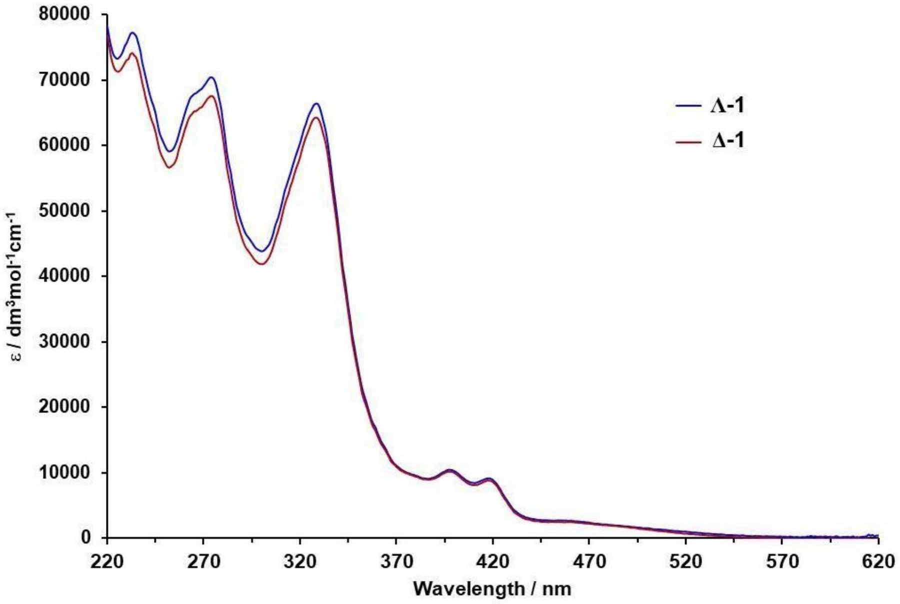

Figure S3 UV-Visible electronic absorption spectra recorded for the resolved Λ- and Δ-enantiomers of **1** in aqueous solution.

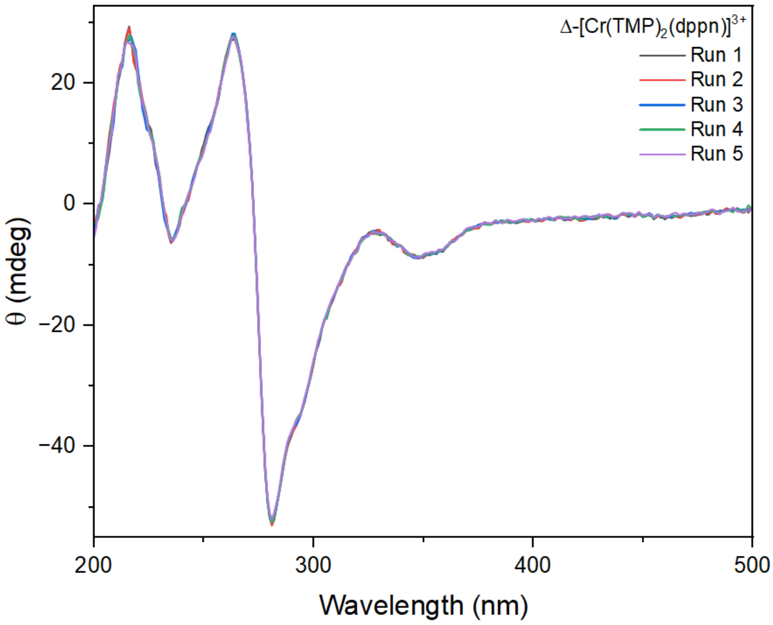

Figure S4: CD spectra of Δ-$[Cr(TMP)_2(dppn)]^{3+}$, with each run consisting of 4 accumulations (20 total). The runs were performed sequentially one after another, no degradation or racemisation was observed under the CD illumination conditions.

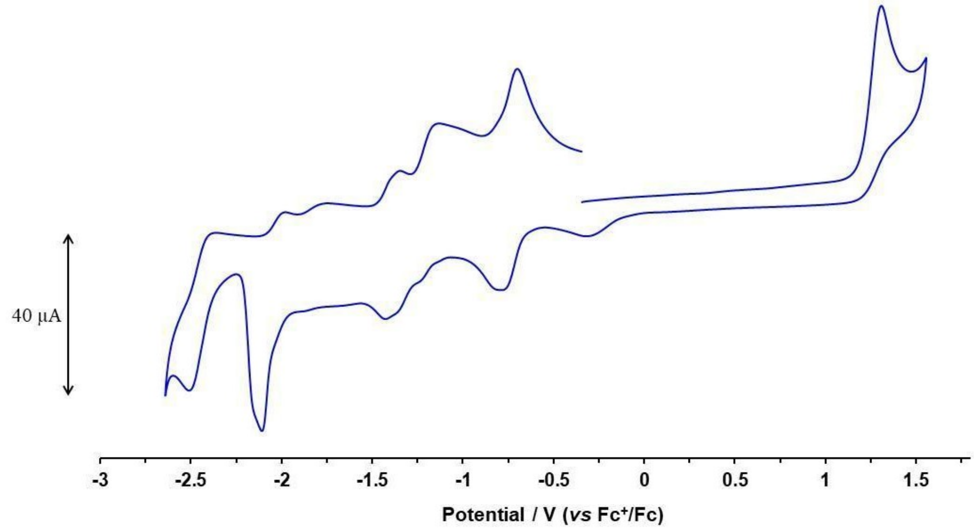

Figure S5 Cyclic voltammogram recorded at 100 mVs^{-1} for a 1.4 mmoldm^{-3} room temperature acetonitrile solution of **1** containing 0.2 moldm^{-3} $^{n}Bu_4NPF_6$ as supporting electrolyte. Potentials are quoted relative to the Fc$^+$/Fc couple.

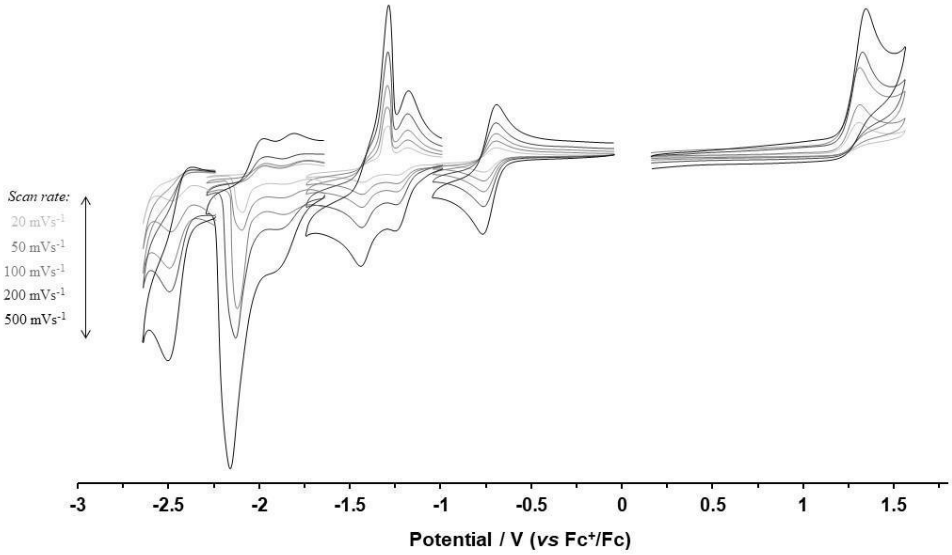

Figure S6 Cyclic voltammetry data recorded for a 1.4 mmoldm^{-3} room temperature acetonitrile solution of **1** containing 0.2 moldm^{-3} $^{n}Bu_4NPF_6$ as supporting electrolyte. Electrochemical processes were scanned at 20, 50 100, 200 and 500 mVs^{-1} in isolation.

Table S1 Summarised electrochemical data for a 1.4 mmoldm^{-3} acetonitrile solution of **1** containing 0.2 moldm^{-3} $^{n}Bu_4NPF_6$ as supporting electrolyte. Measurements were conducted at r.t. at a scan rate of 100 mVs^{-1}. [a] irreversible process, E_p^a; [b] Quasi-Reversible; [c] irreversible process, E_p^c.

	**E½ / V *vs* Fc⁺/Fc ($\Delta E_{a,c}$ / mV)**
**Oxidation**	+1.31[a]
**Reduction**	-0.72 (65), -1.21 (54), -1.39 (79)[b], -2.12[c], -2.45 (116)[b]

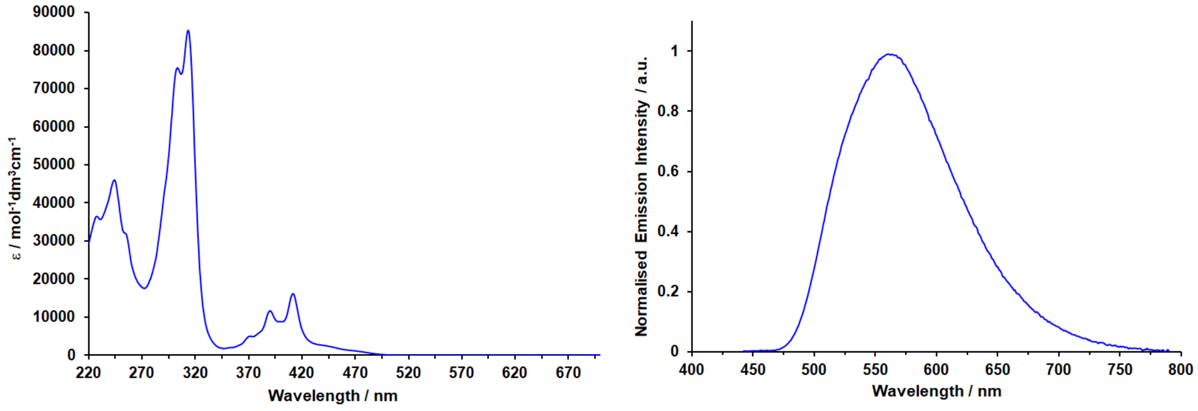

Figure S7 Left: UV-Visible electronic absorption spectrum of free dppn in MeCN solution; Right: Photoluminescence spectrum (λ_{ex} = 400 nm) recorded for an aerated MeCN solution of free dppn at room temperature.

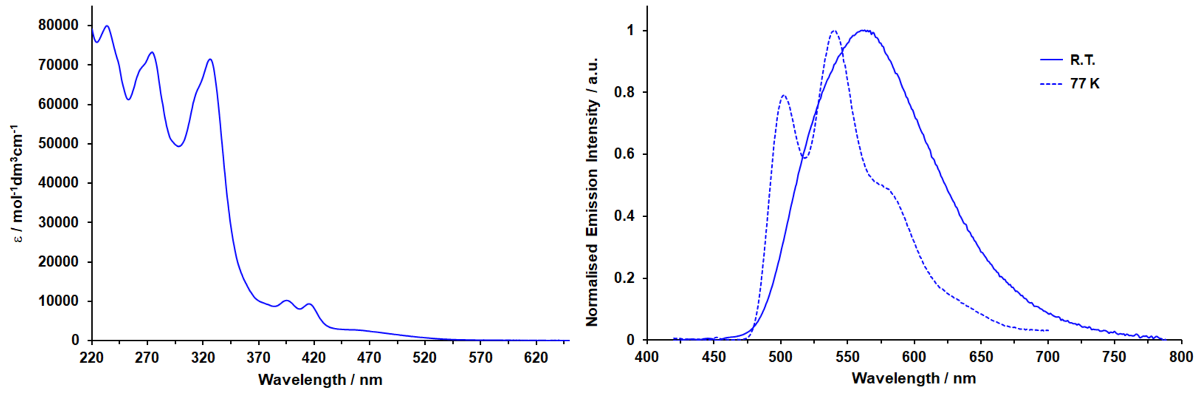

Figure S8 Left: UV-Visible electronic absorption spectrum of **1** in MeCN solution; Right: Photoluminescence spectra (λ_{ex} = 400 nm) recorded for **1** in aerated MeCN solution at r.t. (solid line) and at 77 K in a 4:1 (v/v) EtOH:MeOH glass (dashed line).

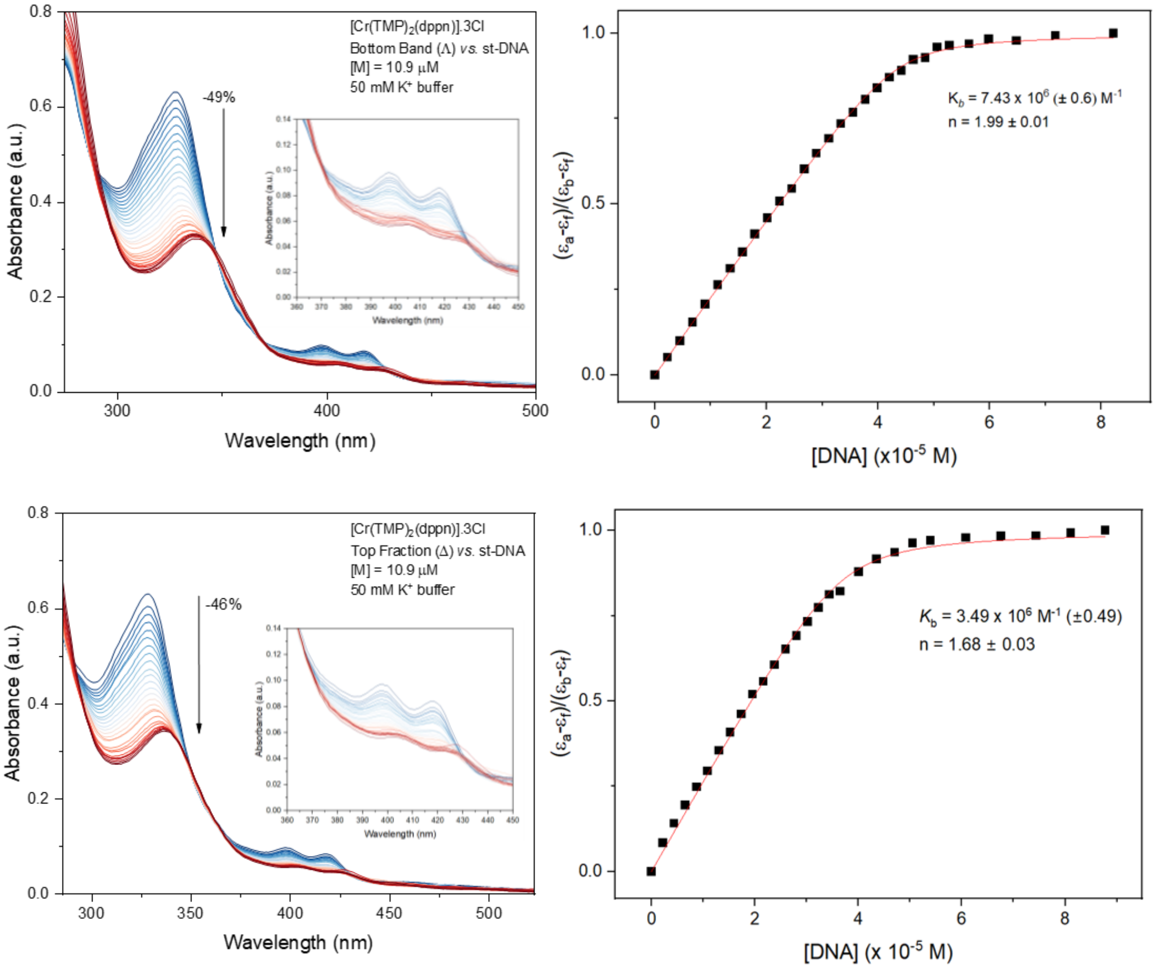

Figure S9: DNA-binding titrations for Λ-**1** (top) and Δ-**1** (bottom) (11 μM) enantiomers *vs.* st-DNA (0-88 μM nucleotide) in 50 mM phosphate buffer.

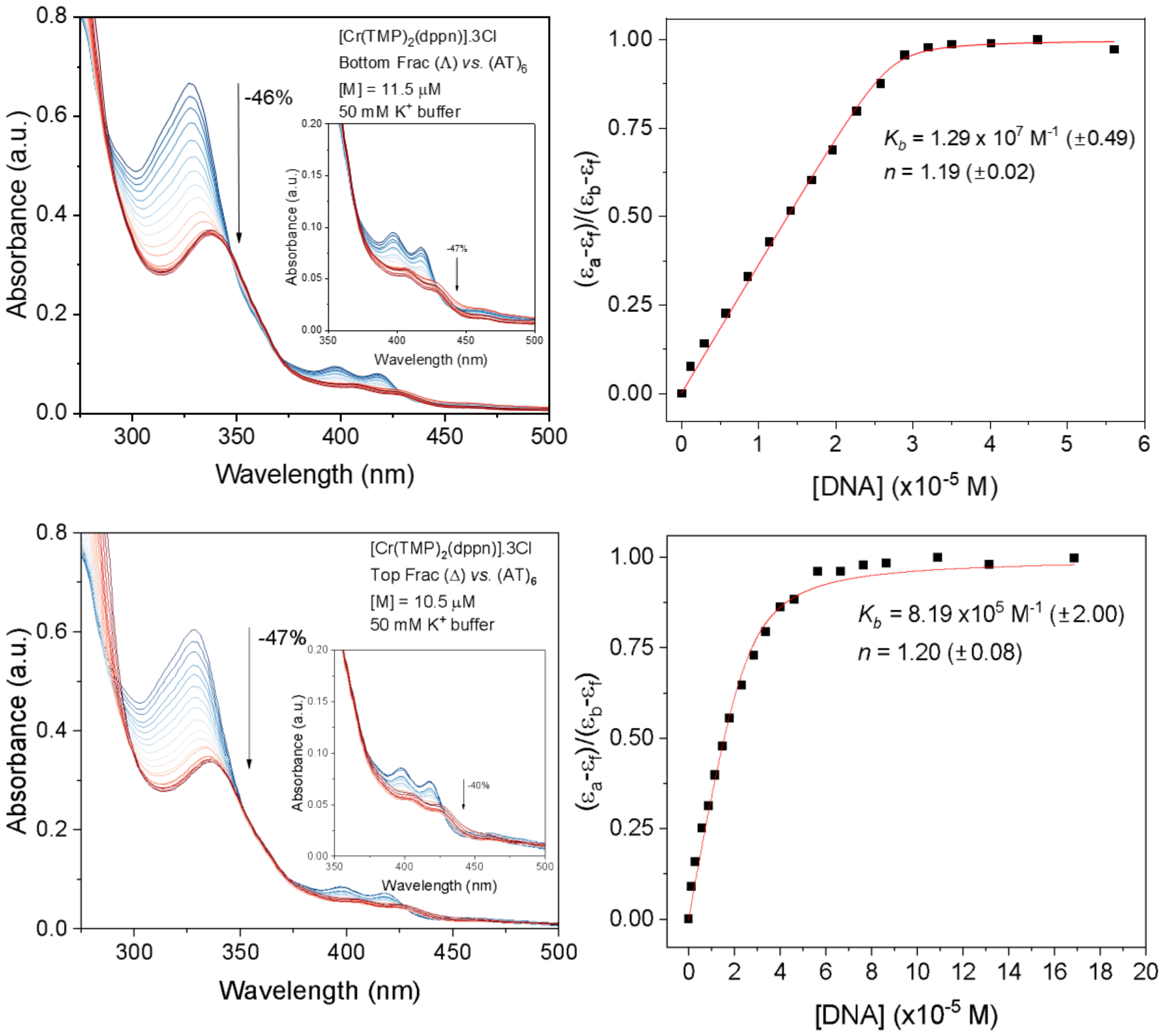

Figure S10: DNA-binding titrations for $[Cr(TMP)_2(dppn)].3Cl$ enantiomers (10.5 μM) *vs.* $(AT)_6$ (0 – 181 μM nucleotide).

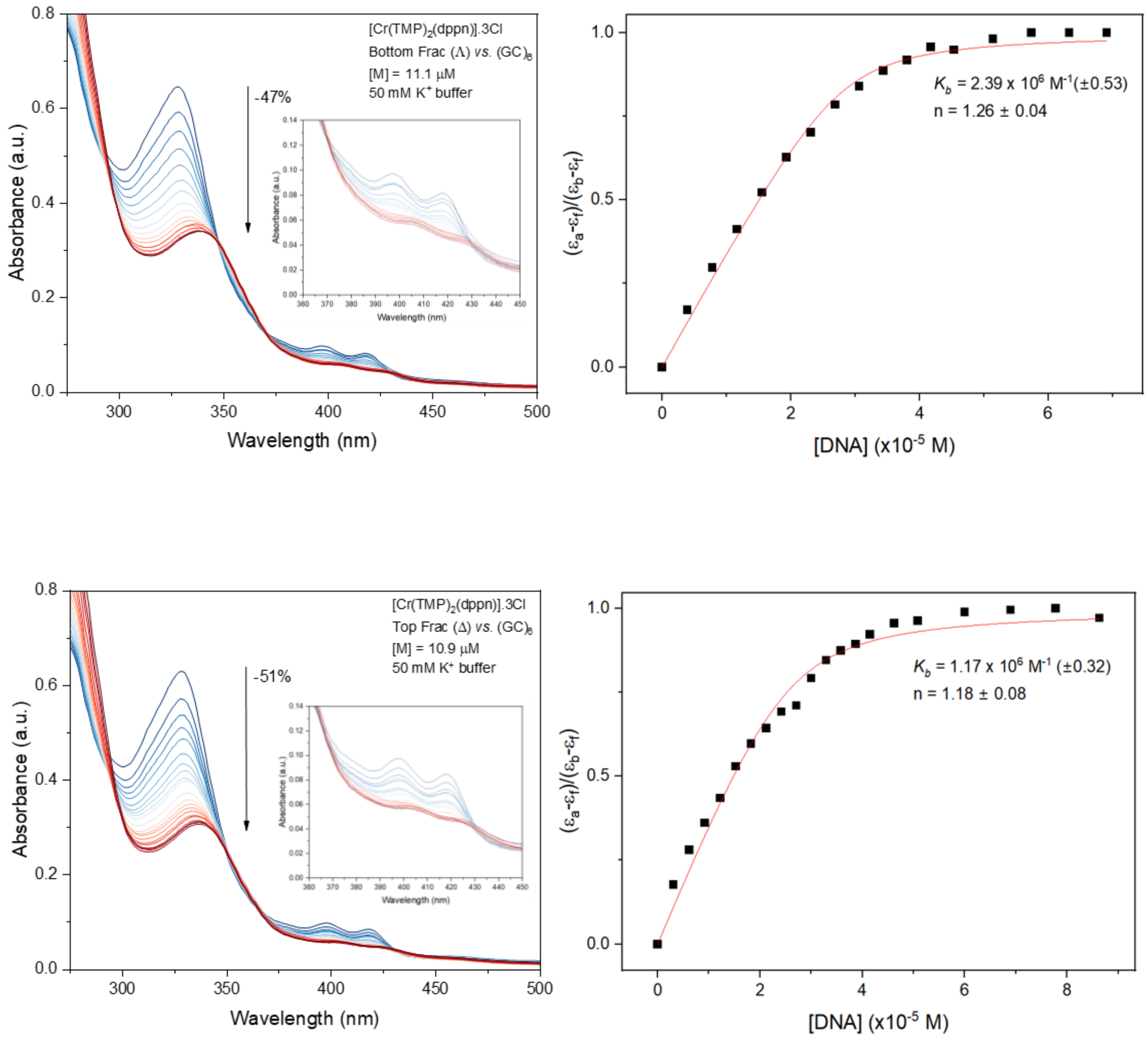

Figure S11: DNA-binding titrations for [Cr(TMP)$_2$(dppn)].3Cl enantiomers (11 μM) *vs.* (GC)$_6$ (0 – 87 μM nucleotide).

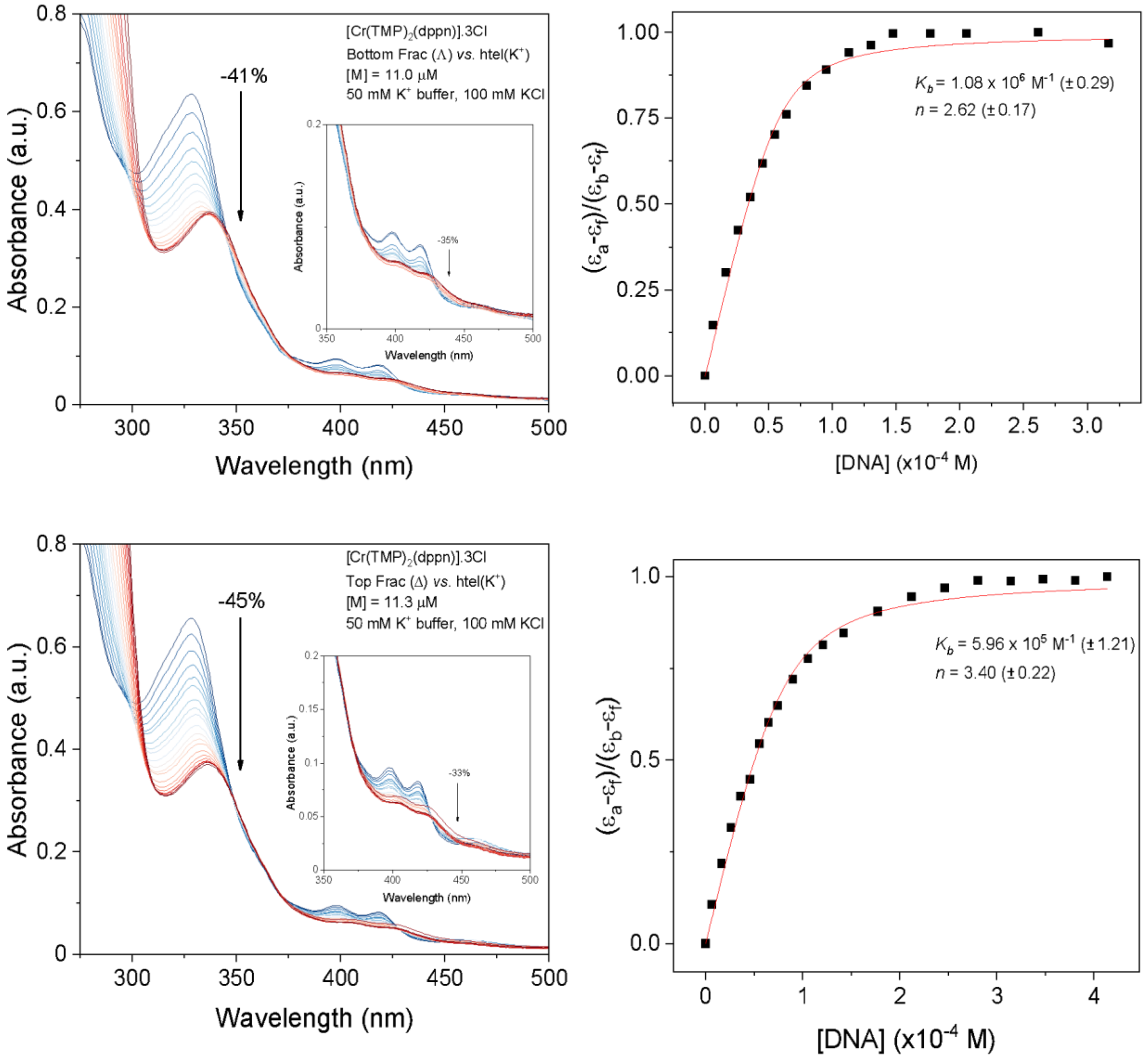

Figure S12: DNA-binding titrations for [Cr(TMP)$_2$(dppn)].3Cl enantiomers *vs.* hTel(K) (0-414 μM nucleotide).

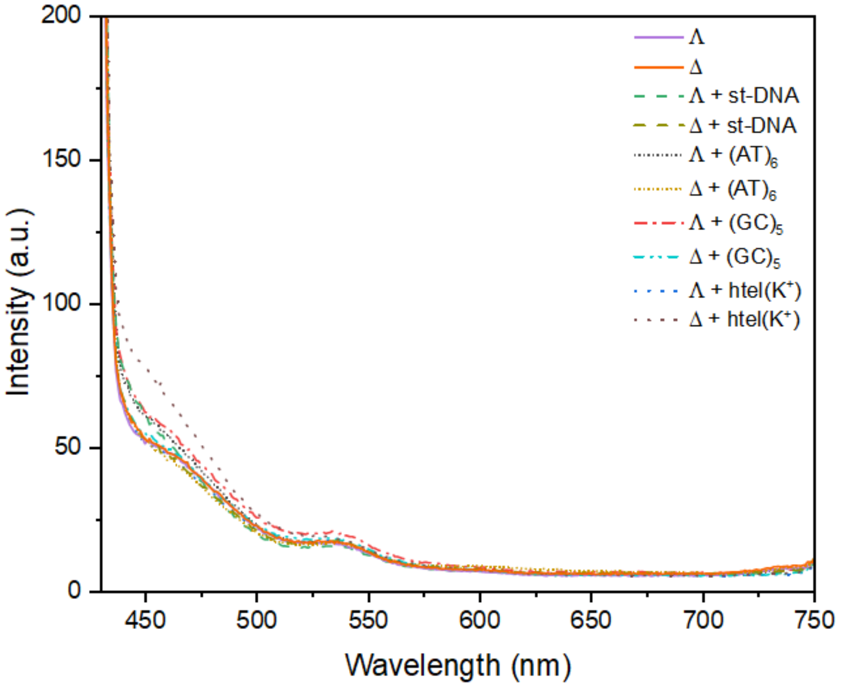

Figure S13: Emission spectra of the enantiomers of $[Cr(TMP)_2(dppn)].3Cl$ in the presence of st-DNA (P/D = 10) or htel(K+) (P/D = 30). Further additions of DNA resulted in no hypochromism in the absorption spectra, indicating full binding under these conditions. No changes in the emission spectra were observed.

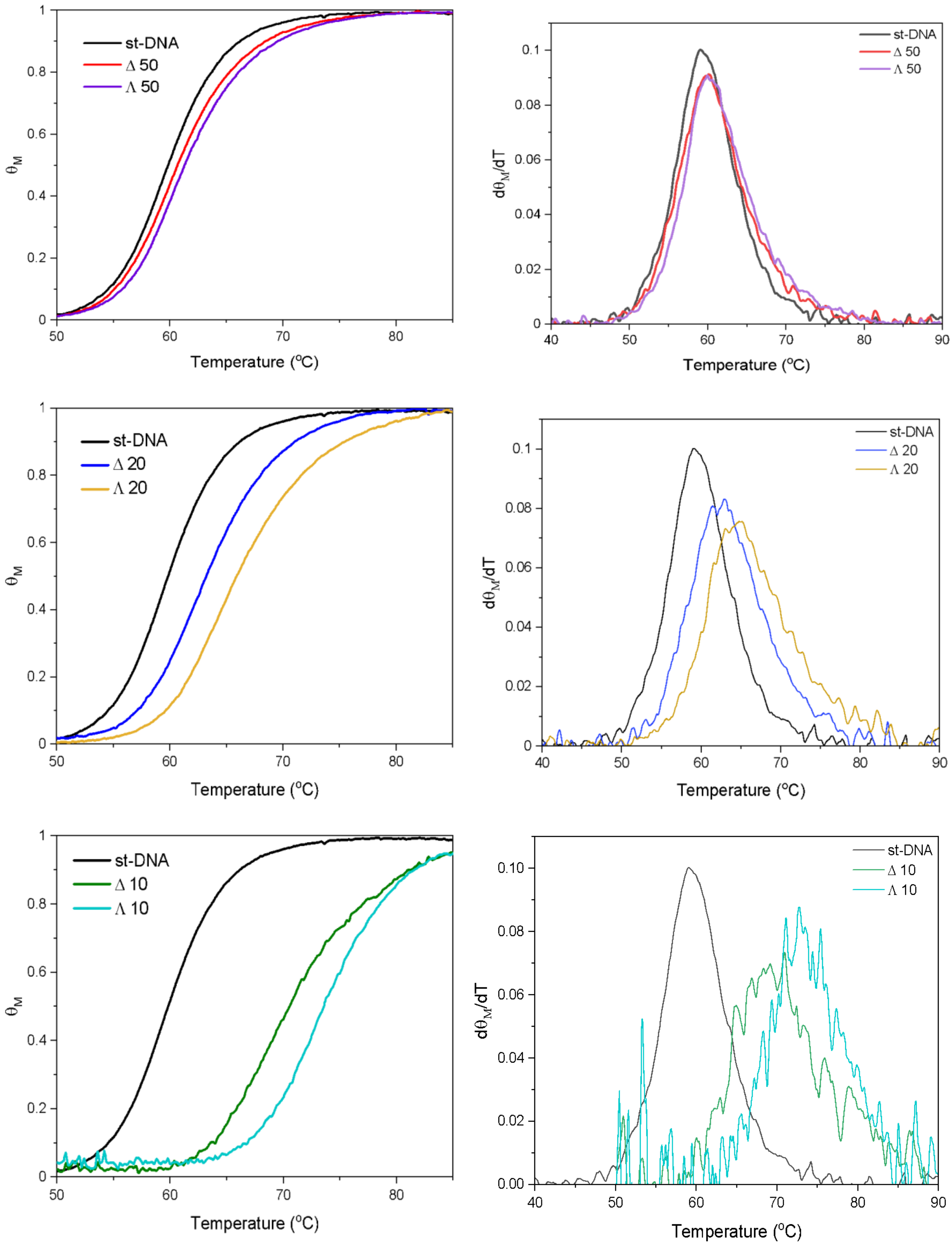

Figure S14: Comparative plots at each P/D value of melting curves and first derivative plots for thermal denaturation of st-DNA in the presence of Λ-**1** and Δ-**1**. Savitsky-Golay smoothing applied to derivative curves in Origin.

References

1. Baptista, F. A.; Krizsan, D.; Stitch, M.; Sazanovich, I. V.; Clark, I. P.; Towrie, M.; Long, C.; Martinez-Fernandez, L.; Improta, R.; Kane-Maguire, N. A. P.; et al. Adenine Radical Cation Formation by a Ligand-Centered Excited State of an Intercalated Chromium Polypyridyl Complex Leads to Enhanced DNA Photo-oxidation. *J. Am. Chem. Soc.* **2021**, *143*, 14766-14779.
2. Carter, M. T.; Rodriguez, M.; Bard, A. J. Voltammetric studies of the interaction of metal chelates with DNA. 2. Tris-chelated complexes of cobalt(III) and iron(II) with 1,10-phenanthroline and 2,2'-bipyridine. *J. Am. Chem. Soc.* **1989**, *111*, 8901-8911.
